# In silico study on probing atomistic insights into structural stability and tensile properties of Fe-doped hydroxyapatite single crystals

**DOI:** 10.1038/s41598-022-24904-0

**Published:** 2022-11-29

**Authors:** Subhadip Basu, Shubhadeep Nag, Nihal B. Kottan, Bikramjit Basu

**Affiliations:** 1 Materials Research Centre, Indian Institute of Science, Bangalore, 560012 India; 2 Solid State and Structural Chemistry Unit, Indian Institute of Science, Bangalore, 560012 India; 3 Center for Biosystems Science and Engineering, Indian Institute of Science, Bangalore, 560012 India

**Keywords:** Atomistic models, Biomaterials, Implants

## Abstract

Hydroxyapatite (HA, Ca_10_PO_4_(OH)_2_) is a widely explored material in the experimental domain of biomaterials science, because of its resemblance with natural bone minerals. Specifically, in the bioceramic community, HA doped with multivalent cations (e.g., Mg^2+^, Fe^2+^, Sr^2+^, etc.) has been extensively investigated in the last few decades. Experimental research largely established the critical role of dopant content on mechanical and biocompatibility properties. The plethora of experimental measurements of mechanical response on doped HA is based on compression or indentation testing of polycrystalline materials. Such measurements, and more importantly the computational predictions of mechanical properties of single crystalline (doped) HA are scarce. On that premise, the present study aims to build atomistic models of Fe^2+^-doped HA with varying Fe content (10, 20, 30, and 40 mol%) and to explore their uniaxial tensile response, by means of molecular dynamics (MD) simulation. In the equilibrated unit cell structures, Ca(1) sites were found to be energetically favourable for Fe^2+^ substitution. The local distribution of Fe^2+^ ions significantly affects the atomic partial charge distribution and chemical symmetry surrounding the functional groups, and such signatures are found in the MD analyzed IR spectra. The significant decrease in the intensity of the IR bands found in the Fe-doped HA together with band splitting, because of the symmetry changes in the crystal structure. Another important objective of this work is to computationally predict the mechanical response of doped HA in their single crystal format. An interesting observation is that the elastic anisotropy of undoped HA was not compromised with Fe-doping. Tensile strength (TS) is systematically reduced in doped HA with Fe^2+^ dopant content and a decrease in TS with temperature can be attributed to the increased thermal agitation of atoms at elevated temperatures. The physics of the tensile response was rationalized in terms of the strain dependent changes in covalent/ionic bond framework (Ca–P distance, P–O bond strain, O–P–O angular strain, O–H bond distance). Further, the dynamic changes in covalent bond network were energetically analyzed by calculating the changes in O–H and P–O bond vibrational energy. Summarizing, the current work establishes our foundational understanding of the atomistic phenomena involved in the structural stability and tensile response of Fe-doped HA single crystals.

## Introduction

Hydroxyapatite (HA,Ca_10_PO_4_(OH)_2_) is the principal inorganic phase in natural bone tissue^1,2^. It is interspersed in the collagen matrix to form a composite structure, and such composite architecture allows the natural bone to withstand the loads experienced by the surrounding tissue^3^. In view of its resemblance with the inorganic composition of the natural bone, HA is widely used to develop synthetic grafts and implants, aimed toward hard tissue regeneration application^4–7^. HA can be artificially synthesized with different Ca/P ratios. The porous scaffolds of HA exhibit moderate strength and better integration with the surrounding tissue (osseointegration)^8,9^. For example, the implant surfaces coated with porous hydroxyapatite are reported to have better osseointegration^10^.

While pure HA has been used extensively for implants and coatings, many studies have explored the effects of various dopants on the structure and properties of Hydroxyapatite. Traditionally, the properties of undoped HA were controlled by factors, such as grain size, porosity, surface roughness, etc.^11^. Doping provides the opportunity to modify the chemical and physical properties of HA. Various ions, such as Ca^2+^, PO_4_^3−^, and OH^−^ ions in the apatite crystal structure can be substituted by cations (Fe^2+^/Fe^3+^, Sr^2+^, Mg^2+^, Zn^2+^, Ag^2+^), and anions (Cl^−^, F^−^, CO_3_^−^)^12–17^. Some dopants, like Ag^+^ are used to inhibit bacterial activity^17^. Overall, the incorporation of these dopants, alone or in combination has been shown to improve bioactivity and osseointegration^18,19^. For example, Fe-doped HA, in particular, appears to improve osseointegration, bone regeneration, and hemocompatibility^12,20,21^. The magnetic behavior of iron-doped HA nanoparticles has been utilized in the field of targeted nanomedicine and MRI imaging^22,23^.

For load bearing applications, the bulk modulus of an implant plays a crucial role in its successful integration with surrounding bone tissue. Stress shielding occurs in a situation, wherein the modulus of the implant material is greater than the surrounding tissue^24,25^. The implant with the higher modulus tends to transfer much less load to the surrounding tissue. This leads to resorption of the bone tissue around the implant, eventually leading to implant failure. While doping undoubtedly improves biocompatibility and specific mechanical properties^26,27^, it is important to study the extent to which doping may improve these properties. While mechanical testing of the polycrystalline doped HA-based bioceramics has been accomplished in many lab-scale studies, the in silico prediction of the mechanical properties of the single crystalline doped HA can have many advantages, and such advantages are relatively unexplored^28,29^.

Molecular dynamics is an in-silico simulation method for studying the temporal evolution of atoms in molecular systems. It employs Newton’s equations of motion to predict the trajectory of various atoms in a system, and allows one to determine the interactions, that atoms have with each other in a given system^30^. MD simulations and DFT calculations have been conducted to study the mechanical properties of HA crystals in a few studies^31–34^. For example, Qin et al. found that HA crystals with sizes up to 2 nm in a collagen fibril matrix can increase mechanical strength^31^. Using the polymer consistent forcefield (PCFF) interatomic potentials, they studied the mechanical behavior of monoclinic HA crystals under different strain rates^32^. Their study indicated anisotropic mechanical response, with the Z direction being the weakest. In compression, the application of strain in the Z direction resulted in better fracture resistance than that in X or Y direction^32^. The temperature was not found to significantly influence the mechanical behavior of the HA crystal. In a different study, Synder et al. studied nanoscale mechanical deformations in hexagonal hydroxyapatite using MD simulations^33^. While using a consistent valence force field (CVFF), they too found that the fracture strength under tension was weakest along Z axis and strongest under compression. While comparing the mechanical properties of systems containing 44,000 atoms and 5500 atoms, they found very little variation in properties.

While the mechanical properties of pure HA have been studied from a molecular dynamics perspective, few studies have explored the mechanical properties of doped HA^35,36^. Particularly, there are no molecular dynamics studies exploring the effect of iron doping in HA, and its mechanical response at different temperatures. As HA experiences different ranges of temperature, during manufacturing, sterilization, and implantation, it becomes important to study the temperature-dependent and compositional-dependent mechanical behaviour of Fe-doped HA. Against the above backdrop, this study aims to perform a molecular dynamics study on iron-doped HA, specifically to find the changes in the FTIR spectra and the mechanical properties in comparison to pure HA. Also, brittle materials, like HA are more susceptible to failure under tension and exhibit much better properties under compression. We present here the results obtained under tensile loading of undoped /doped HA single crystals, when strained along X, Y, or Z axes.

## Methods

**Figure 1:**
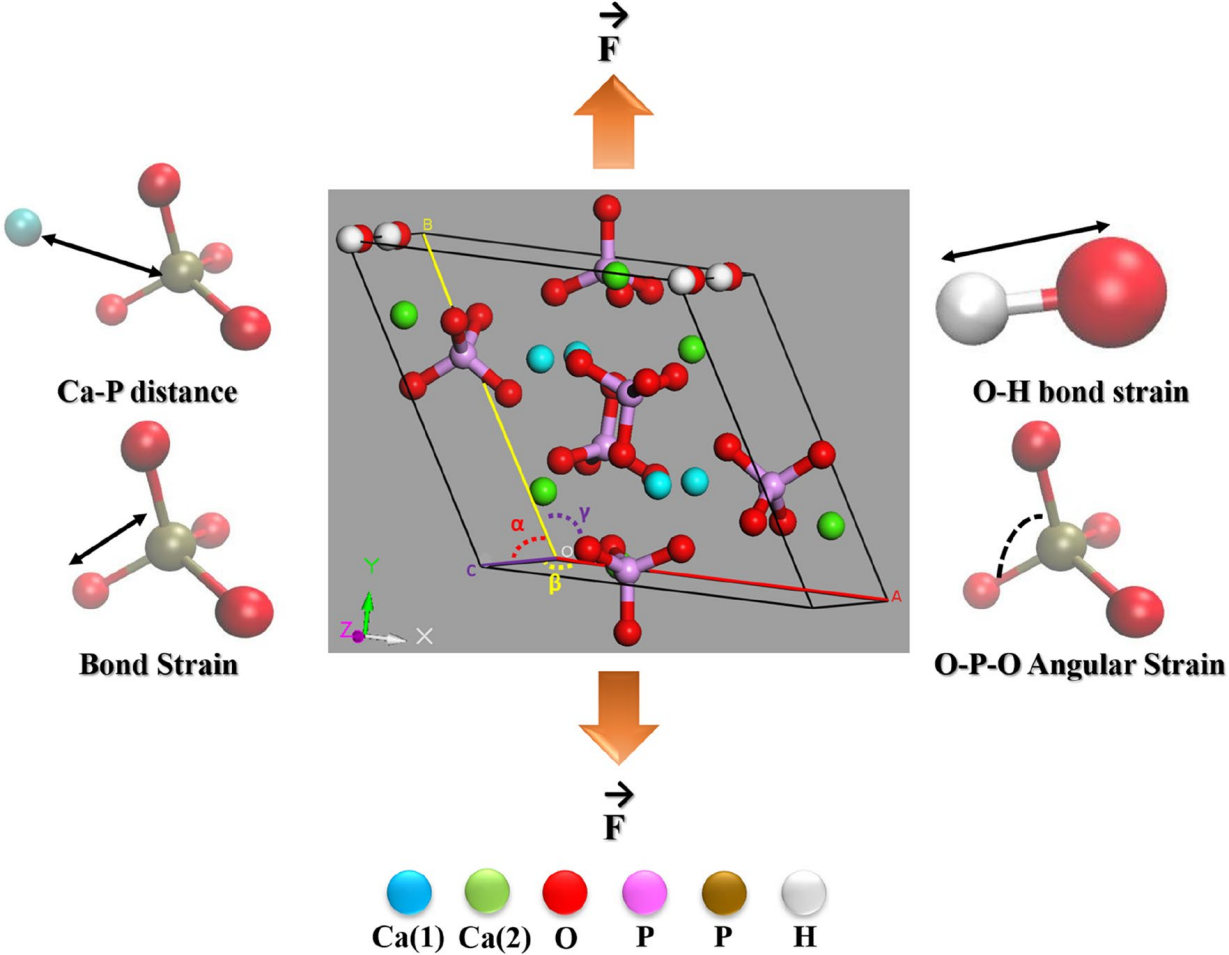
Schematic illustration of different aspects of the covalent/ionic bond framework during tensile loading, as analyzed using MD simulation. The unit cell of undoped hydroxyapatite (HA), with minimum energy configuration is obtained using Materials Studio 6.0. The crystallographic parameters are shown with colour code: *a, α:* red, *b, β:* yellow, *c, γ:* Violet.

The unit cell of HA consists of 44 atoms with hexagonal symmetry. The lattice parameters are described as a = 9.417 Å, b = 9.417 Å, and c = 6.875 Å, with interior angles α = β = 90° and γ = 120°^37,38^. In the present study. HA unit cell was modelled using BIOVIA Materials Studio 6.0^39^. Figure 1 shows a ball and stick representation of the structure. A supercell consisting of 1180 atoms was created for the study of the mechanical response. The structure was then geometrically optimized using the smart algorithm, implemented in Materials Studio package^40^. The optimized structure was then equilibrated first at a desired temperature for 100 ps. This was followed by a second phase of equilibration at 1 atmospheric pressure for 100 picoseconds. Berendsen thermostat and barostat were employed for temperature and pressure coupling, respectively^41^. The cell time constant was kept at 1.0 ps and the decay constant was kept at 0.1 ps.

### In silico tensile response analysis

The equilibrated structure was used in LAMMPS to evaluate the uniaxial tensile response, in silico^42^. The PCFF-interface forcefield was used to conduct the simulations^43^. Earlier studies have reported that the ‘Interface Forcefield’ provides reasonable accuracy in determining bulk modulus^32,33^. The tensile loading simulations were performed using a model of 1188 atoms. The periodic boundary conditions were applied in all 3 directions. The cutoff distance for Lennard–Jones and Coulombic pair potentials was set to 9.5 Å, similar to the study by Snyder et al.^33^. Ewald summation was used in all simulations, to ensure the acceptable convergence of the system^44^. On MD simulation platform, the system was uniaxially and virtually loaded in the X, Y, and Z directions at 10 K, 300 K, and 400 K to study the tensile properties. The rationale for the selection of different temperatures can be explained as follows. Since we have used different force field equations for undoped and Fe-doped HA, we wanted to initially assess the reliability of our results against the published results with undoped HA at 10 K^32^. During synthesis, HA or doped HA experiences sub-zero temperatures during freeze-drying. For bioactivity assessment, HA/doped HA is tested for biomineralization at 300 K. Similarly, prior to biocompatibility testing, HA-based materials undergo steam autoclave-based sterilization at 400 K. Therefore, it was important to understand the impact of such variation in temperature on interatomic bond configuration changes as well as the mechanical response under tension, which primarily causes the failure of brittle ceramics like HA at much lower stress or strain values, compared to that under compression. Also, a few published papers reported the compression failure of undoped HA single crystals, using MD simulations and therefore, the present in silico study is expected to add new results on HA system^33^.

In each case, the system was first equilibrated at the respective temperature for 100 picoseconds before the load application. A strain rate of 1 × 10^10^/s was applied in each case, to study the stress response of the structure. This was then used to derive the tensile properties of the system.

### In silico structural analysis

The IR spectra analysis was carried out using LAMMPS and available python code^42,45^. LAMMPS was first used to calculate the net dipole moment of the system. A python script was then used to calculate the dipole–dipole autocorrelation function and to perform the Fourier transform to obtain the IR line shape. The IR spectra were then calculated by multiplying the lineshape with an electromagnetic field-dependent prefactor.

The Fourier Transform was used to calculate the IR lineshape, which is given by the following equations:

1 $$I\left( \omega \right) = \frac{1}{2\pi } \mathop \smallint \limits_{ - \infty }^{\infty } e^{{ - i\left( {\omega - \left( \omega \right)} \right)t}} \phi \left( t \right)dt$$

2 $${\text{Where}},\quad \phi \left( t \right) = \left\langle {\vec{\mu }_{{01}} \left( 0 \right).\vec{\mu }_{{01}} \left( t \right)e^{{i\mathop {{{ \int}}}\limits_{0}^{t} \delta \omega \left( \tau \right)d\tau }} } \right\rangle e^{{ - \left| t \right|/2T_{1} }}$$

Here, $\delta \omega \left( t \right) = \omega \left( t \right) - \langle\omega\rangle$, $\vec{\mu }_{01} \left( t \right) = \vec{\mu }_{01} \left( t \right){\vec{\text{e}}}_{OH} \left( t \right)$ is the $0 \to 1$ (fundamental) transition dipole vector for the OH oscillator directed along the bond, and *T*_*1*_ is the vibrational energy relaxation time.

In order to study the Fe doped hydroxyapatite structure, the Ca^2+^ ion was replaced with Fe^2+^ ion in either Ca(1) position or in Ca(2) position in the HA unit cell. Energy minimization was performed on both the structures to acquire the most stable structures together with an insight into preferable substitution sites of Fe^2+^ ions in apatite structure. The stable structures thus obtained were subjected to mechanical testing and spectral analysis, following the above-mentioned protocols. Throughout the text, xFeHA designates × mol% Fe-doped HA and the sample designations are mentioned in Table 1.

**Table 1:** Lattice parameters and interior angles for the unit cell of Fe-doped HA structure.

Structure	Designation	a = b (Calculated) (Å)	a = b (Experimental) (Å)	Deviation(a_cal_ − a_exp_)/a_exp_ × 100	c (calculated) (Å)	c (Experimental) (Å)	Deviation (c_cal_ − c_exp_)/c_exp_ × 100	α (°)	β (°)	γ (°)
0 mol% Fe-HA	0FeHA	9.417	9.413	0.04	6.875	6.880	− 0.06	90	90	120
10 mol% Fe-HA	10FeHA	9.614	9.417	2.09	7.200	6.887	4.55	90	90	120
20 mol% Fe-HA	20FeHA	9.669	9.418	2.66	7.247	6.887	5.22	90	90	120
30 mol% Fe-HA	30FeHA	9.803	9.422	4.05	7.197	6.900	4.35	89.99	90	120
40 mol% Fe-HA	40FeHA	9.939	9.400	5.74	7.148	6.893	3.71	90	89.99	120

Experimental values are taken from our previous study^46^. In the table, a_exp_/c_exp_ denotes experimental values of lattice parameters, and a_cal_/c_cal_ denotes calculated lattice parameters.

## Results

We shall analyze the results in two important aspects. First, the IR spectrum will be calculated on the basis of the structural parameters as well as the unit cell configuration for different composition of Fe-doped Hydroxyapatite. The second aim is to understand the anisotropy in the mechanical response as a function of Fe^2+^ content. Furthermore, the effects of temperature on mechanical properties will also be quantitatively assessed.

### Insight into phase stability in Fe-doped HA single crystals

**Figure 2:**
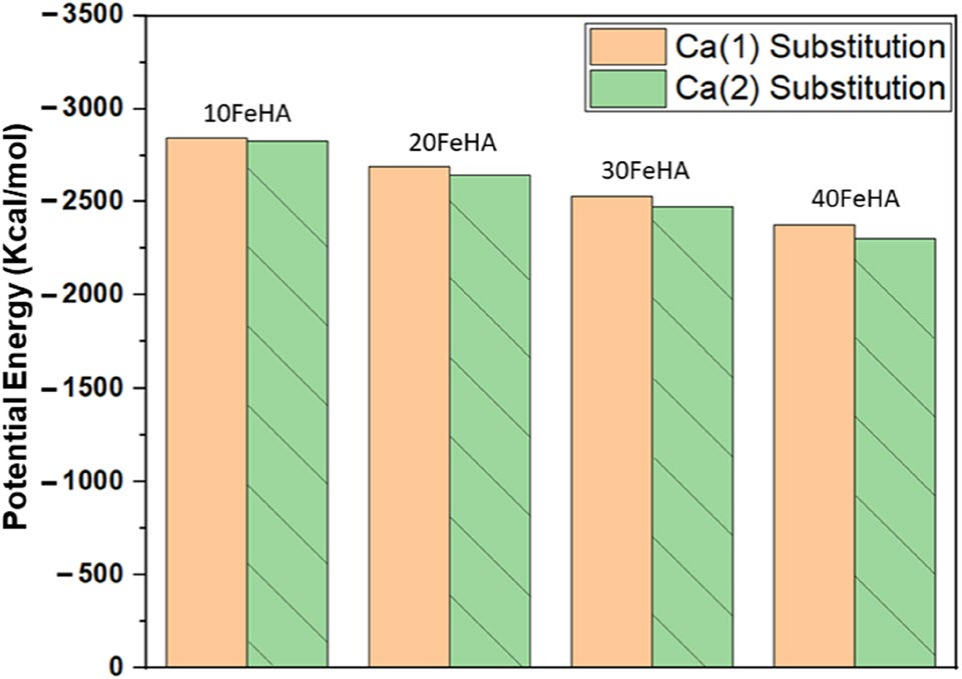
Minimum potential energy of doped HA structures, as determined using Materials Studio, based on the calcium substitution site. (Sample designation: xFeHA means  x mol% Fe^2+^-doped HA).

**Figure 3:**
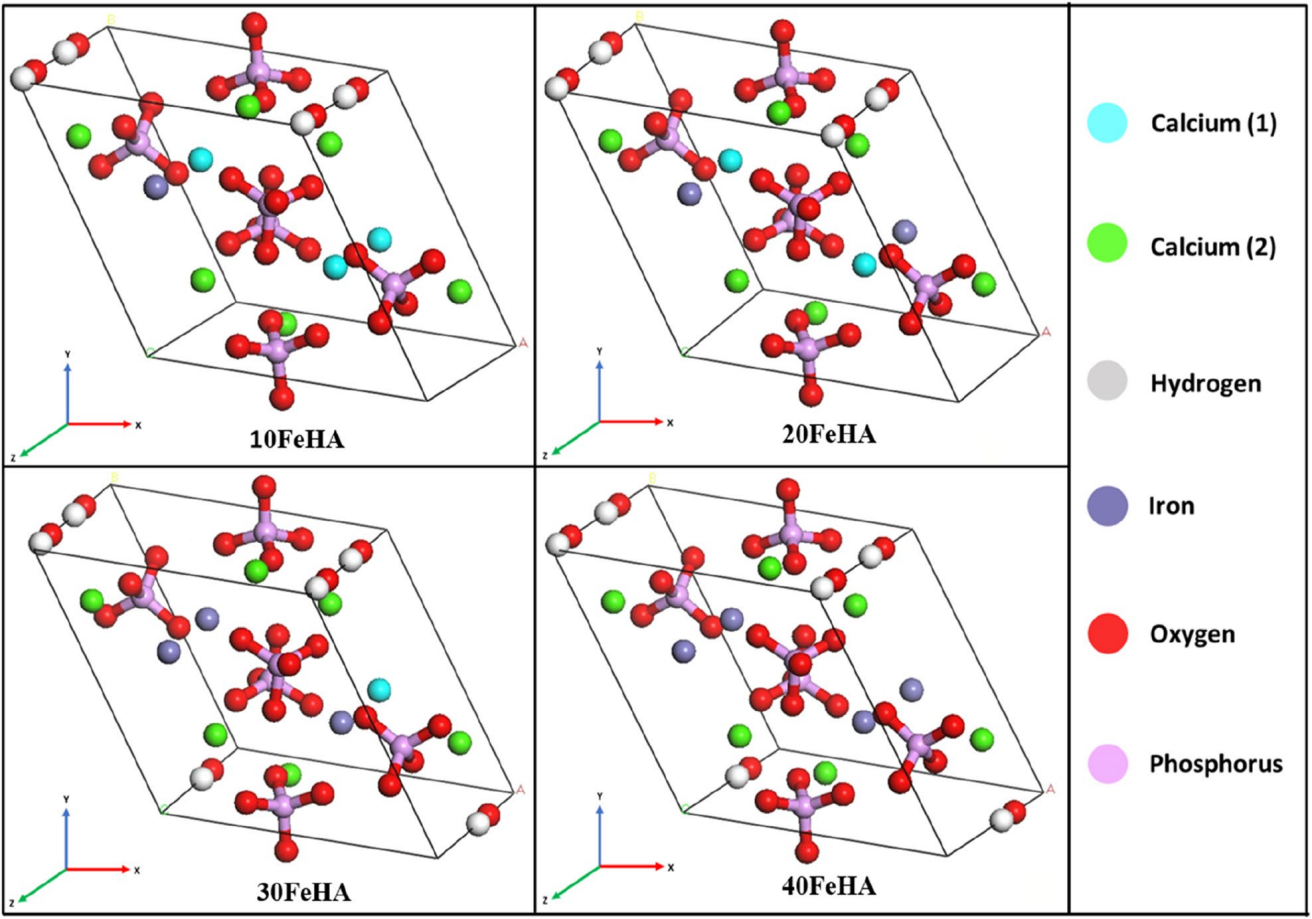
Equilibrated unit cell structure of different Fe-doped HA. 10FeHA, 20FeHA and 30FeHA contain heterogenous atomic columns along ‘*c*’ axis, where Ca^2+^ and Fe^2+^ reside adjacent to each other. (Sample designation: xFeHA means x mol% Fe^2+^-doped HA).

The initial structure of HA unit cell has been presented in Fig. 1. Primarily, the difference in energy states between the Fe^2+^ substituted structures at Ca(1) and Ca(2) positions were explored, and the final energy values, obtained after the minimization was depicted in Fig. 2. From Fig. 2, it can be easily deduced that the Ca(1) positions are slightly favourable for Fe^2+^ substitution, irrespective of the dopant content, probably because of the smaller volume of Ca(1) site^35^. Another important aspect is the stability of the apatite phase after Fe^2+^ doping. It can be clearly seen from Fig. 2 that, the minimum energy of the HA phase systematically decreased with an increment in Fe^2+^ content. In harmony with the experimental evidences, the substitution of Fe^2+^ ions negatively impacts the phase stability of HA^47^. The energetically minimized structures (Fe^2+^ doped at Ca(1) positions) were shown in Fig. 3. All further calculations were carried out using the structures doped in Ca(1) positions, as shown in Fig. 3.

The substitution of Fe^2+^ results in the change in different crystallographic parameters and those are summarized in Table 1. A systematic increase in lattice parameters *a* and *b* with Fe^2+^ content has been observed, while a fluctuating trend was noticed along ‘c’-axis (Table 1). For instance, lattice parameter (*a)* increased from 9.417 Å in pure HA to 9.669 Å in 20FeHA (Table 1). A slight reduction (9.939 Å) was observed in 40FeHA (Table 1). For the lattice parameter (*c*), it first increased from 6.875 Å in pure HA to 7.247 Å in 20FeHA and again reduced to 7.148 Å in 40FeHA (Table 1). It is interesting to note that with Fe^2+^ substitution, the ‘*c’* axis changes most significantly compared to ‘*a’* and ‘*b’* (Table 1). For example, the change in *a* in 40FeHA was only 0.5% compared to pure HA, whereas the change in the* c* axis was found to be 3.9%.

Now, we shall compare the energetically minimized structural data of Fe-doped CaPs, as discussed above, with the experimentally measured data, reported in our previous study, wherein the lattice parameters of Fe-doped biphasic calcium phosphate, BCP (a mixture of HA and β-TCP) were determined using XRD-based Rietveld refinement method^46^. In most of the cases, the theoretically calculated values are overestimated compared to the experimental values (Table 1). The observed deviations can be attributed to the following reasons.

Firstly, the synthesized materials were polycrystalline in nature, and a second phase (β-TCP) was present alongside HA^46^. Secondly, there is a difference between the oxidation states of Fe dopant ions. A better comparison would have been possible if the lattice parameters of single-crystalline Fe-doped HA are available. On the other hand, lattice angles remain almost constant with very marginal changes (Table 1). In our experimental work, the lattice angles were found to be conserved^46^. Hence, there are no major distortions in the lattice structure. This reconfirms the well-known ability of apatite structure to retain its crystal symmetry after doping^35^.

### Spectroscopical analysis

**Figure 4:**
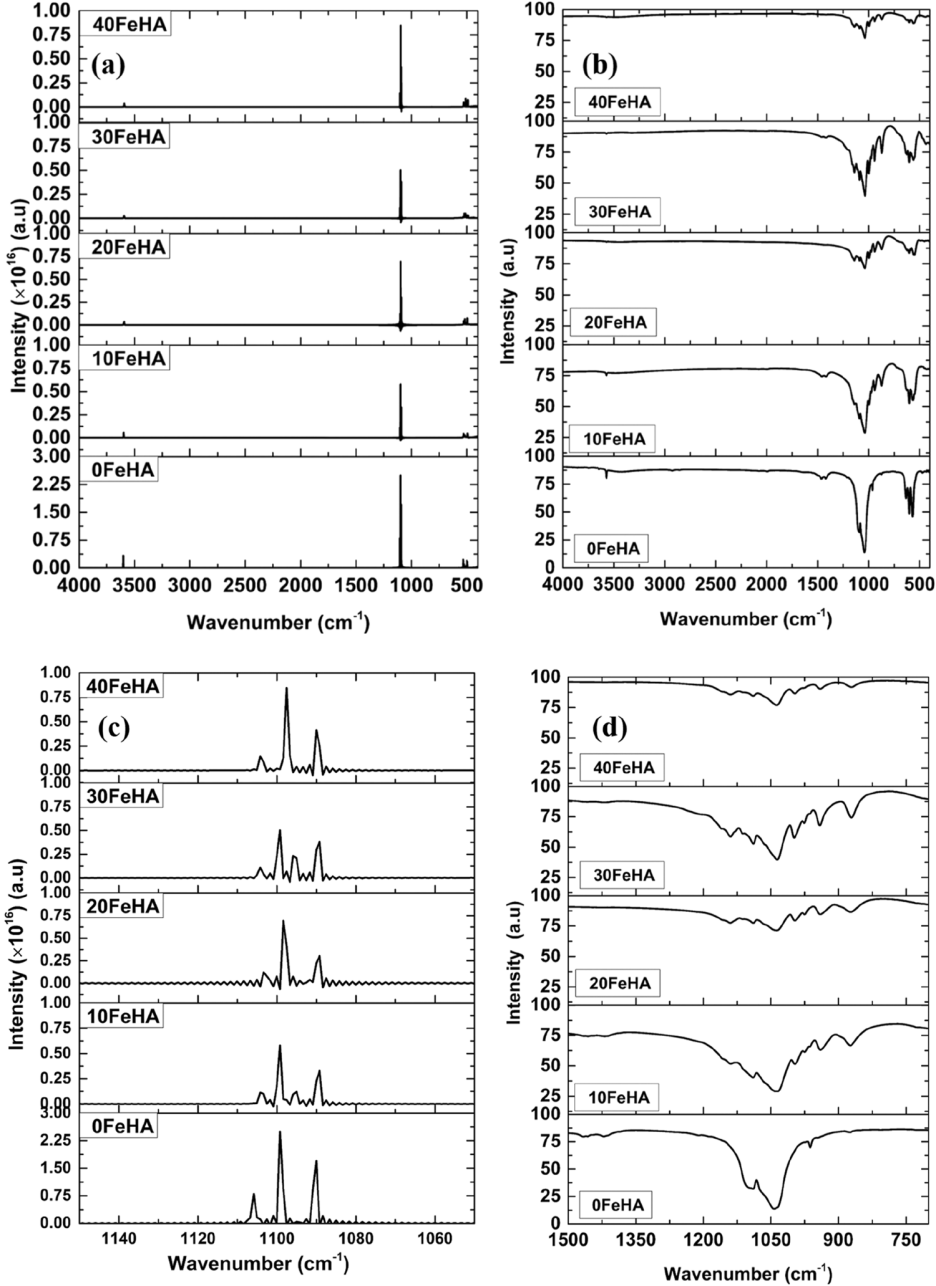

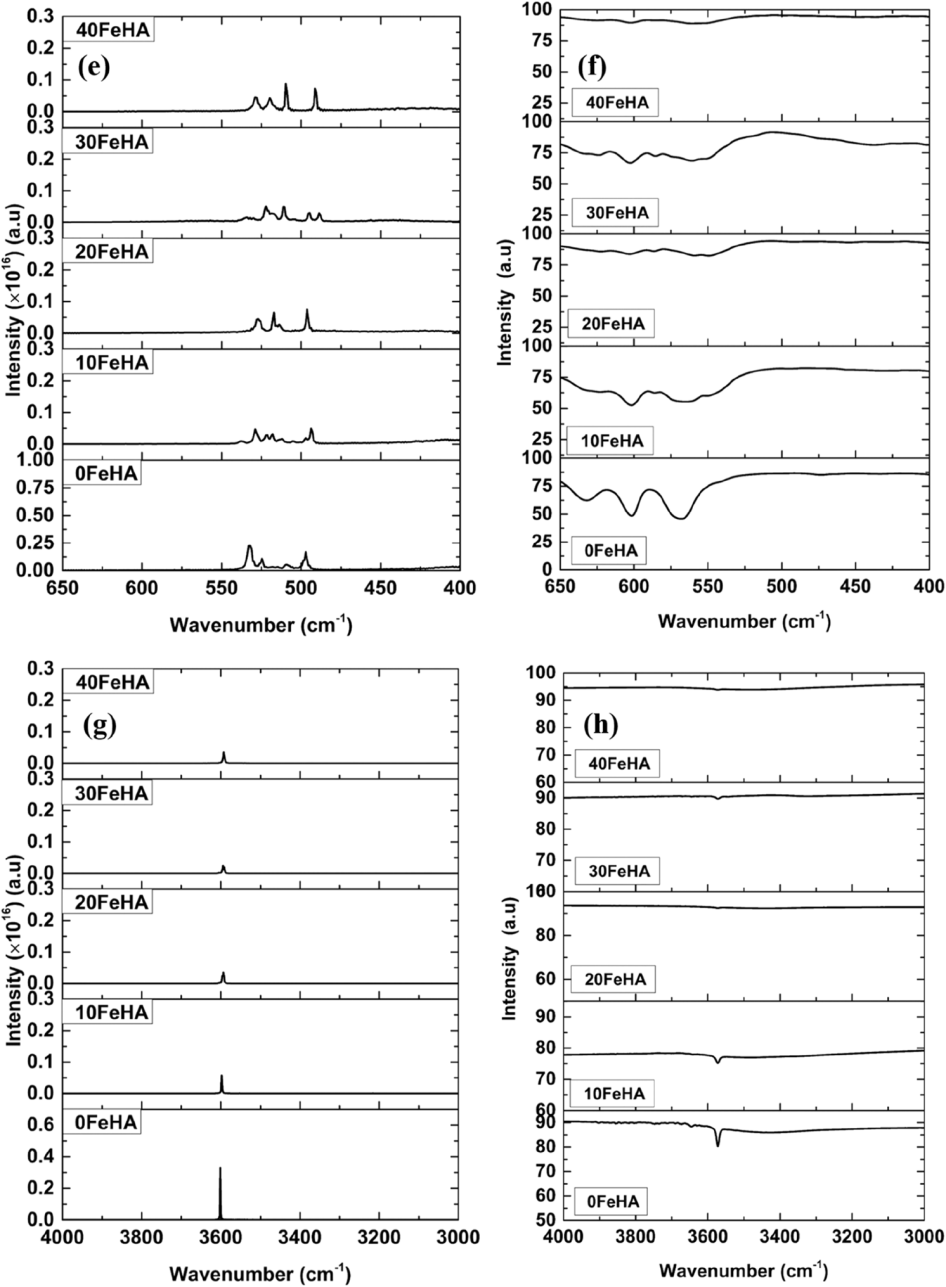
(**a**) Calculated and (**b**) experimental IR spectra different Fe-doped HA in the range of 4000–400 cm^−1^. (**c**) Enlarged views of calculated IR spectra between the range of 1150–1050 cm^−1^ and (**d**) Experimental IR spectra in the range of 1500–700 cm^−1^. A larger area has been enlarged in (**d**) because of the experimental band broadening. Magnified views of (**e**) computed, and (**f**) experimental IR spectra in the range of 650–400 cm^−1^ Bands of (**g**) simulated and (**h**) experimental IR spectra in the range of 4000–3000 cm^−1^ are also presented. Experimental spectra are taken from our previous study^46^. (Sample designation: xFeHA means x mol% Fe^2+^-doped HA).

The calculated IR spectra of undoped and different Fe^2+^ doped HA have been calculated from the dipole–dipole autocorrelation function and have been presented in Fig. 4. For the sake of comparison, the experimental IR spectra of Fe-doped BCP, from our earlier experimental study have also been shown in Fig. 4^46^. The band positions of different modes of vibrations of various functional groups are tabulated in Table S1 (see supplementary information). It is evident from Fig. 4 and Table S1 that the IR band positions of the calculated spectra of pure HA corroborate well with the experimental results^46,48^. The most prominent IR bands in the calculated spectra in the region of 1090–1110 cm^−1^ correspond to the asymmetric stretching mode (*v*_3_ ) of the phosphate group (Fig. 4c)^48^. Moreover, small bands, corresponding to the symmetric bending modes (*v*_2_) and *v*_4_ bands of the phosphate groups can also be seen in the region of 497 cm^−1^ and 532 cm^−1^, respectively (Fig. 4e). In addition, the IR band at ~ 3600 cm^−1^ represents the hydroxyl stretching (Fig. 4g).

The number and the intensity of the IR bands were found to be affected by the Fe^2+^ doping into the apatite structure. The most notable change appeared in the (*v*_3_) vibrational mode of the phosphate group (Fig. 4). It can be easily seen that the intensity of the (*v*_3_) bands was greatly reduced with the Fe^2+^ doping and the lowest intensity was found for 10 and 30 mol % Fe-doped HA (Fig. 4). Together with such observations, the splitting of the most prominent IR bands, leading to the appearance of extra bands at ~ 1095 cm^−1^, was also recorded for 10 and 30 mol% Fe-doped HA (Fig. 4). These results endorsed well with the experimental observations, where band splitting can be seen in Fe-doped samples (Fig. 4b,d). On the other hand, the reduced intensity of OH^−^ vibrational bands at ~ 3500 cm^−1^ with increasing Fe-content can be observed in both computed and experimental IR spectra (Fig. 4g,h).

The calculated change of the IR spectra can be explained in the qualitative manner. Among several factors, the number and intensity of the IR bands depend on the symmetry surrounding the functional group and on the partial charge distribution of the atoms. The presence of symmetry helps to reduce the number of bands. With the substitution of Ca^2+^ ions by Fe^2+^ ions, it is observed that the symmetry around the phosphate groups along with the partial charge distribution significantly changed, as seen in Fig. 2 and Table 3. It is evident from Fig. 3 that, the symmetry surrounding the phosphate group was most affected in the case of 10 and 30 mol% of Fe^2+^ doping. This breakage of symmetry caused the reduction in the intensity together with the splitting of the *v*_3_ vibrational bands in two Fe-doped HA.

### Uniaxial tensile response of single crystals of Fe-doped HA

MD simulation was successfully implemented to predict the mechanical stress–strain response under tension. As mentioned earlier, we have conducted simulations at three different temperatures namely 10 K, 300 K, and 400 K. We have first benchmarked the simulation results at 10 K with the results published in the literature by Snyder et al. and Ou et al.^32,33^. A comparison of the simulation results with the existing literature shows that the mechanical response of undoped HA along the X, Y, and Z-axis follows a similar pattern. We have considered a system consisting of 1188 atoms to conduct our simulation within a reasonable time period. However, simulations in the existing literature have used larger systems (44,000 atoms)^33^. The usage of this smaller system results in significant undulations in the post-TS stress–strain response. In the current study, an important parameter viz*.* the tensile strength, which represents the maximum load-bearing capacity of the single crystals, has been extracted from the stress–strain response and has been analyzed with respect to both temperature and dopant concentration.

**Figure 5:**
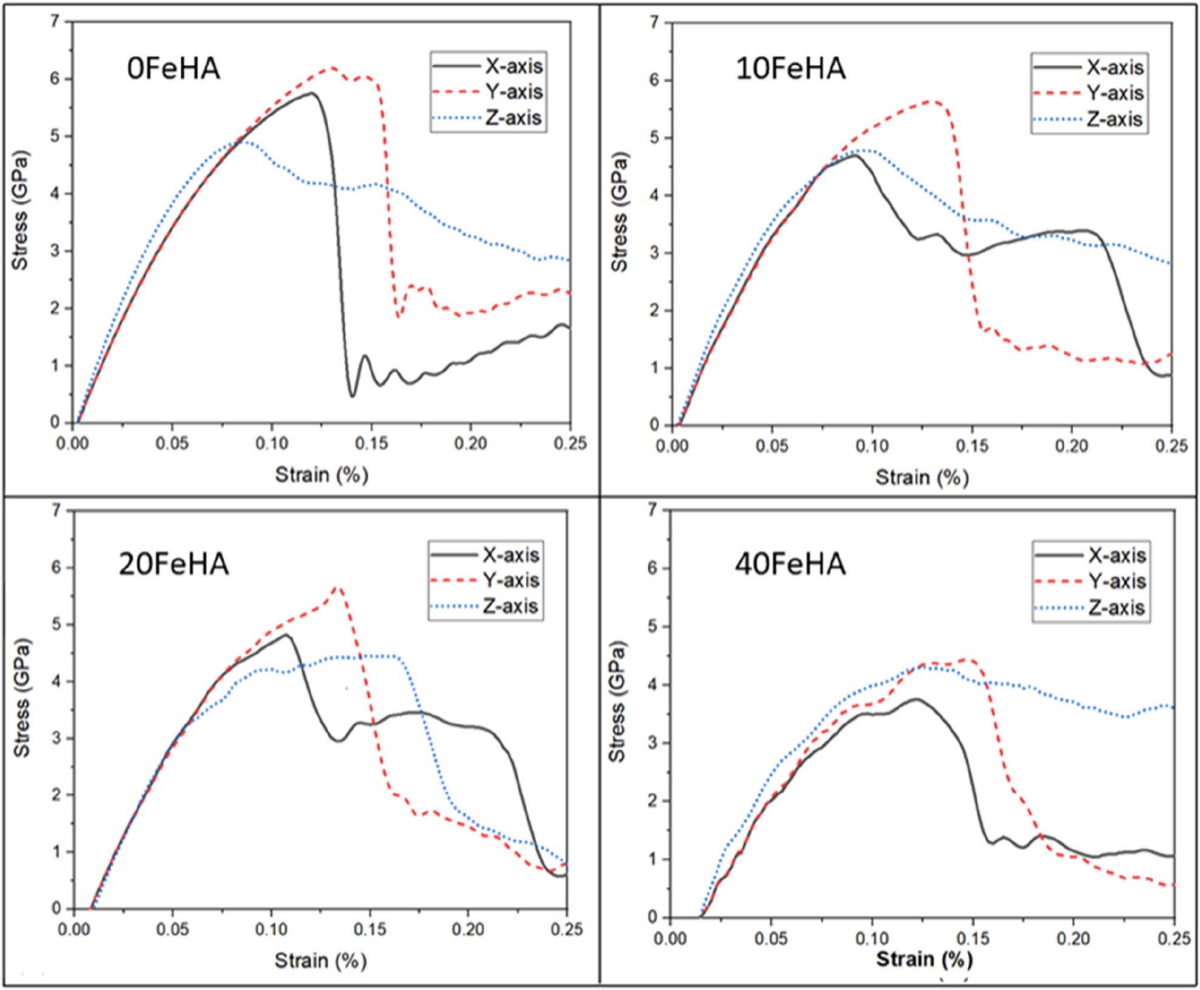
Uniaxial tensile stress–strain response at T = 10 K for different doped HA, as analyzed using MD simulation. (Sample designation: xFeHA means x mol% Fe^2+^-doped HA). The tensile loading directions along different crystallographic orientations follows similar manner as shown in Fig. 1.

Figure 5 shows the stress–strain response of both doped and undoped HA at 10 K. The common characteristic is that the system undergoes a linear response, followed by a nonlinear response. Post yielding, there is a sharp drop in the stress values in almost all the cases, except when the system is strained along Z-axis (Fig. 5). One notable observation is the appearance of two prominent peaks in the stress–strain curve for Fe-doped HA at 10 K, when loading direction was along the X axis (Fig. 5). This can be attributed to the breakage and rearrangement of the ions inside the material during the tensile test. The tensile strengths obtained from these computed stress–strain curves have been tabulated in Table 2. It can be noted that the maximum value of tensile strength (point of maximum stress) was always recorded when the load was applied along Y-direction (Table 2, Fig. 5). The tensile strength also remained lower in doped HA, compared to the pure one at 10 K (Fig. 5, Table 2).

**Table 2:** Tensile strength of systems at different temperatures and dopant concentrations.

Structure designation	Tensile strength (Gpa)								
	T = 10 K			T = 300 K			T = 400 K		
	X-axis	Y-axis	Z-axis	X-axis	Y-Axis	Z-axis	X-axis	Y-axis	Z-axis
0FeHA	5.65	6.20	4.90	4.65	5.50	4.00	4.50	5.05	3.75
10FeHA	4.60	5.60	4.70	4.30	4.75	3.52	4.00	4.50	3.10
20FeHA	4.80	5.60	4.50	4.00	4.60	3.48	3.75	4.35	3.15
40FeHA	3.75	4.50	4.30	3.20	3.70	3.75	3.10	3.50	3.20

**Figure 6:**
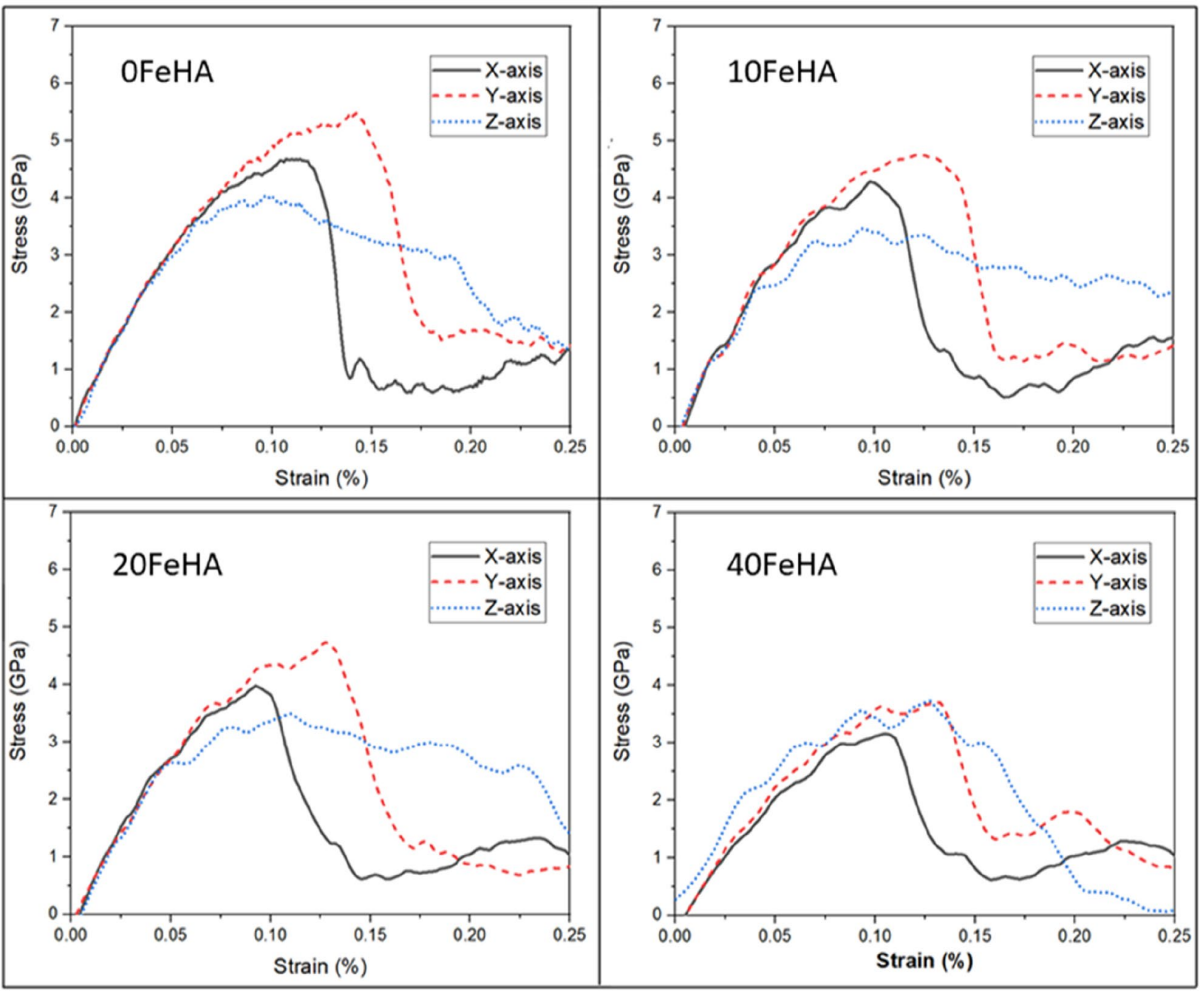
Uniaxial tensile stress–strain response at T = 300 K for different doped HA, as analyzed using MD simulation. (Sample designation: xFeHA means x mol% Fe^2+^-doped HA). The tensile loading directions along different crystallographic orientations follows similar manner as shown in Fig. 1.

**Figure 7:**
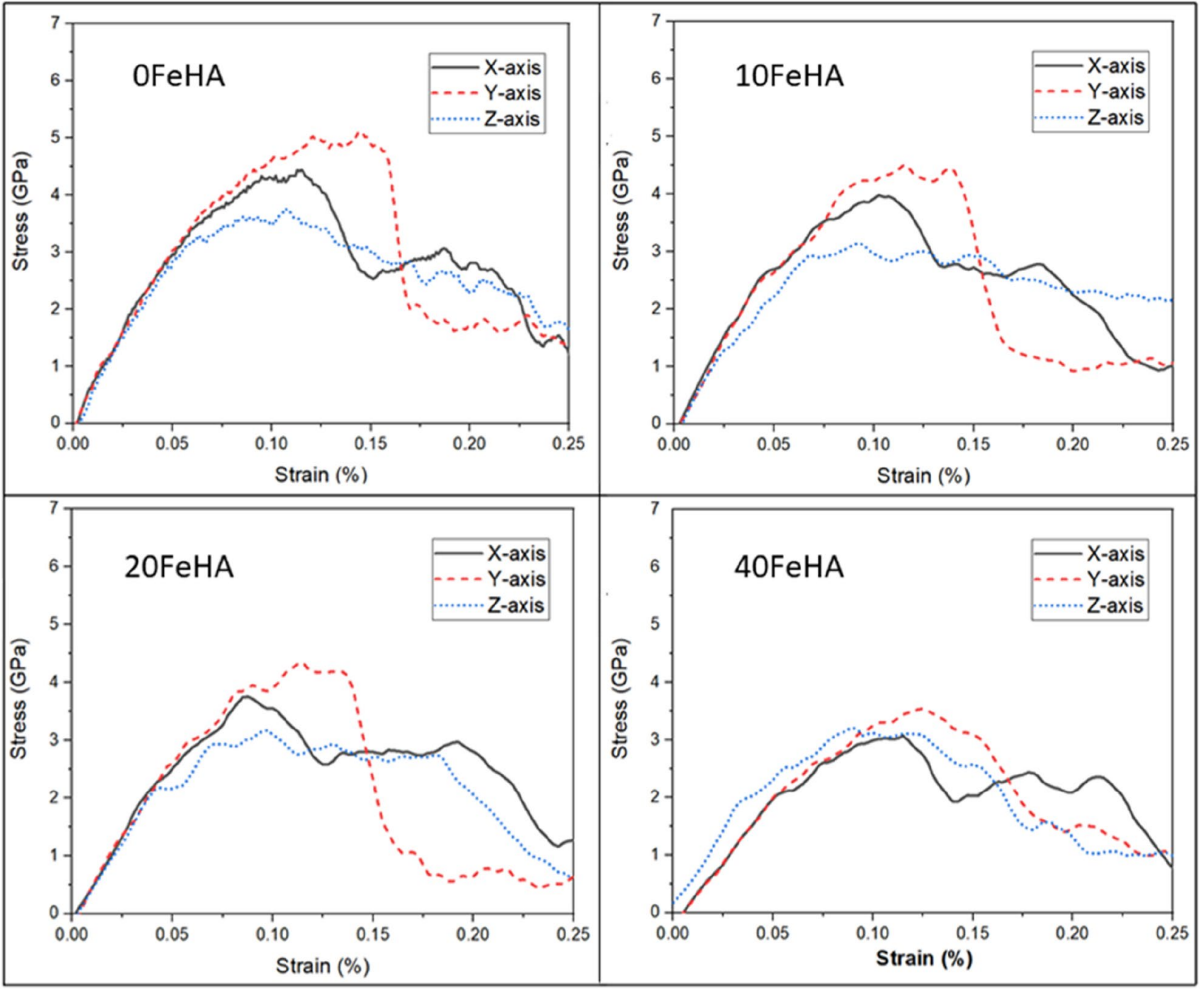
Uniaxial tensile stress–strain at T = 400 K for different doped HA, as analyzed using MD simulation. (Sample designation: xFeHA means x mol% Fe^2+^-doped HA). The tensile loading directions along different crystallographic orientations follows similar manner as shown in Fig. 1.

Similar stress–strain response was recorded at higher temperatures (Figs. 6 and 7). One notable difference is the presence of undulations in the stress–strain curve of pure HA with loading along the X-axis at 400 K (Fig. 7). The values of tensile strength (TS) decreased systematically with an increase of temperature, independent of the loading direction and dopant content (Table 2). In most cases, the TS always remained lower in doped HA than in pure HA (Table 2).

## Discussion

In our efforts to develop a deeper insight into the physics of tensile response, we shall now analyze the atomistic insights and the rearrangement or restructuring of the covalent bonds in doped HA single crystals.

### Atomistic insights into tensile response of single crystalline doped HA

**Figure 8:**
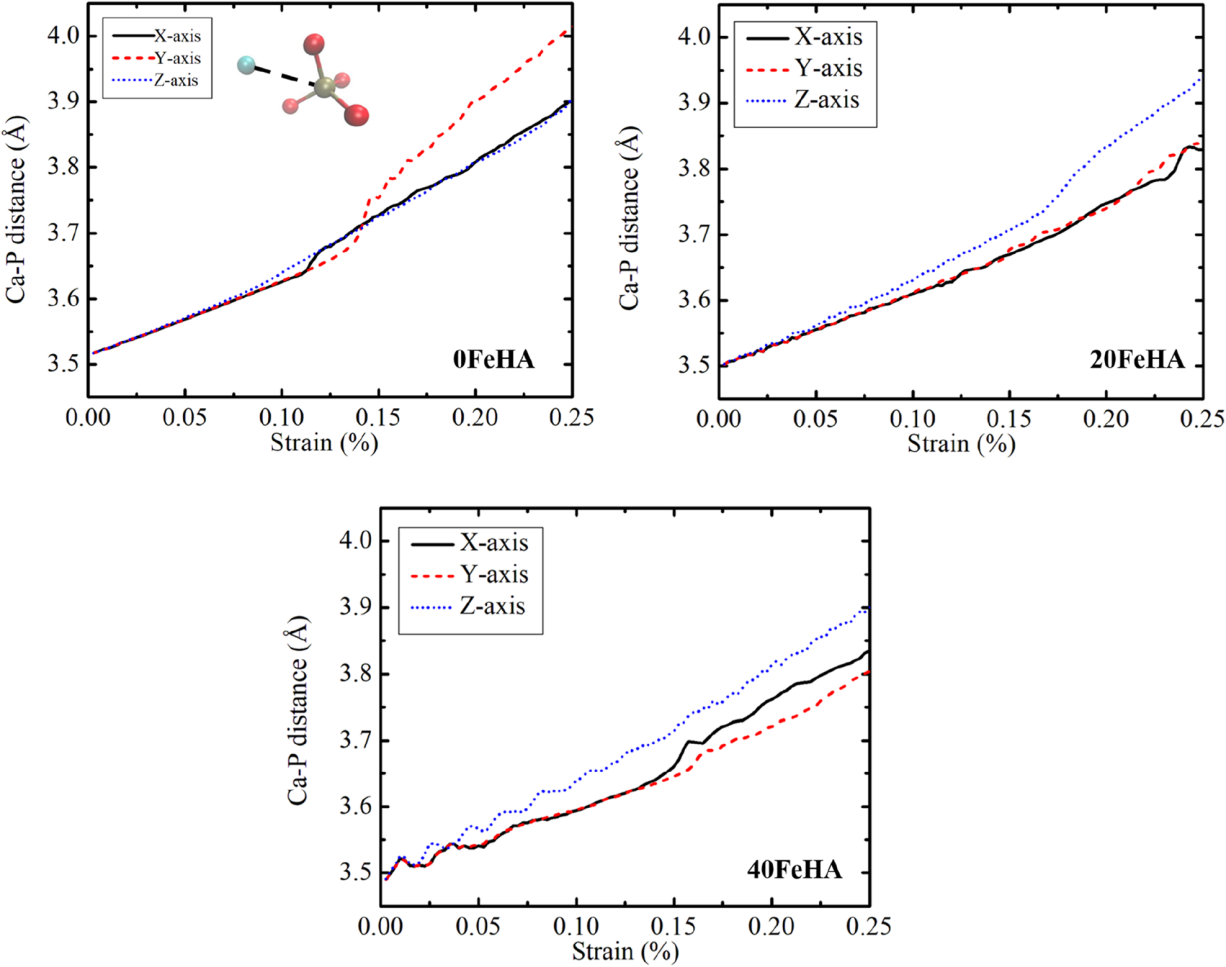
MD simulated dynamic changes in distance between Ca^2+^ and P^5+^ ions with uniaxial tensile strain, for different doped HA at T = 10 K. The distance has been defined schematically in the inset. Atomic colour code: Ca: pale blue, O: red, P: golden-yellow. (Sample designation: xFeHA means x mol% Fe^2+^-doped HA).

**Figure 9:**
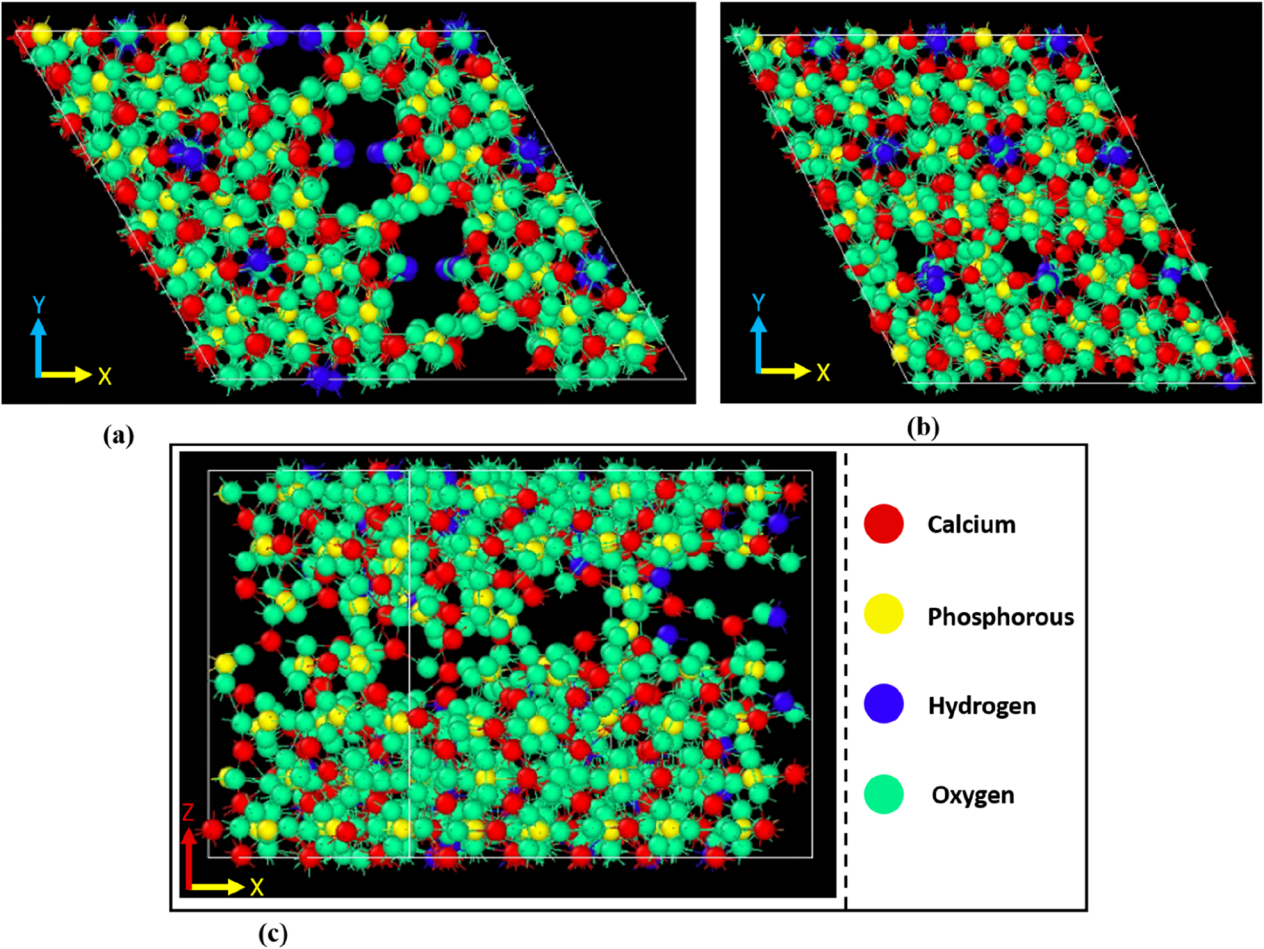
MD simulation acquired snapshot of undoped HA (0FeHA) at T = 300 K at the vicinity of TS (highest point on the stress-strain curve), when load is applied along (**a**) X-axis, (**b**) Y-axis and (**c**) Z-axis. (Sample designation: xFeHA means x mol% Fe^2+^-doped HA).

**Figure 10:**
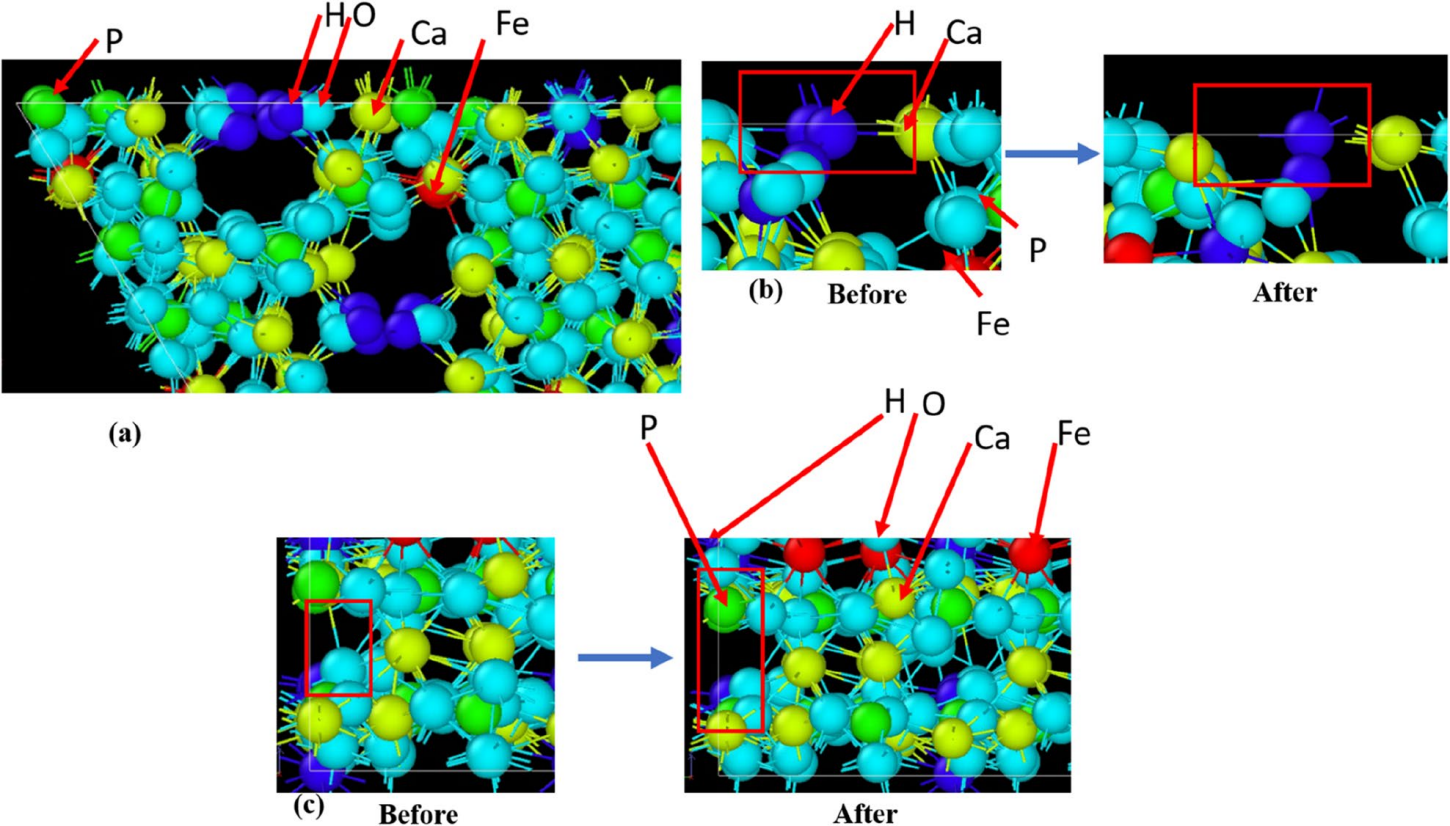
MD simulation acquired snapshot of 10FeHA at T = 300 K at the vicinity of TS (highest point on the stress-strain curve) when loaded  along (**a**) X, (**b**) Y, and (**c**) Z axis. “Before” and “After” indicates the state of the system prior and after bond breaking at TS point. The region(s) marked by red square highlights broken bond(s). (Sample designation: xFeHA means x mol% Fe^2+^-doped HA).

In order to obtain atomistic insight into the stress–strain response, the distance between Ca^2+^/Fe^2+^ and phosphate ions has been calculated. For each case, the distance was found to be monotonously increasing with the applied strain, almost in a linear manner (Fig. 8, Fig. S4 and S5 in supporting information). An interesting observation about the variation in Fe–P distance is the presence of more fluctuation than Ca–P distance, specifically at higher temperatures. Such fluctuating behavior is a signature of the thermal agitation/vibration of the system, which increases with temperature. As Fe^2+^ carries lesser partial charges than Ca^2+^ (Table 3), the thermal perturbation is more prominent in the Fe–P distance compared to the Ca–P distance, due to reduced coulombic attraction. This increment of the ionic distance is the major contributor to the mechanical response of doped HA. Moreover, it is clear from the above trends that the tensile response of undoped and Fe-doped HA strongly depends on the loading direction. Different atomic arrangement along the orthogonal crystallographic directions is the underlying reason behind the observed anisotropic nature of HA. Profoundly, different sets of atoms are displaced in the crystal during the mechanical response, depending on the loading directions. For instance, in the case of pure HA, OH^−^ ions are observed to move apart, during loading along the X-axis (Fig. 9). This indicates the effective reduction of the attractive interaction between OH^−^ ions and Ca^2+^ ions, which ultimately led to material failure. Similarly, the interactions among Ca^2+^ ions and PO_4_^3−^/OH groups were noted to be reduced, when the load was applied along the Y-direction (Fig. 10). As more ionic bonds were broken along Y-direction, the tensile strength was higher along the Y-axis (Table 2).

**Table 3:** Partial charges on different atoms in undoped and Fe-doped HA, calculated using charge equilibration (Qeq) method^49^.

Element name	Partial changes (a.u)				
	0FeHA	10FeHA	20FeHA	30FeHA	40FeHA
Ca	1.210–1.250	1.233–1.272	1.251–1.282	1.269–1.300	1.287
O(H) (Oxygen in OH^−^)	(−)0.837	(−)0.818− (−)0.816	(−)0.796− (−)0.797	(−)0.775− (−)0.776	(−)0.754
O(P) (Oxygen in PO_4_^3−^)	(−)0.562− (−)0.590	(−)0.560− (−)0.601	(−)0.537− (−)0.581	(−)0.546− (−)0.573	(−)0.527− (−)575
P	0.480	0.494–0.499	0.513	0.527–0.533	0.546–0.547
H	0.230	0.236–0.243	0.242–255	0.254–0.261	0.267
Fe		0.735	0.750	0.752–0.773	0.775

On the other hand, the mechanical response of pure HA along the Z-direction strongly depends on the ionic bonds between Ca^2+^ ions and PO_4_^3−^ groups, which started to break during tensile testing (Fig. S2, S3 in Supporting information). The above-mentioned trend is observed independent of the temperature and the doping amount of HA.

Besides this, the redistribution of the atomistic charges in the presence of the foreign metal ions also alters the mechanical behaviour, which has been substantiated further in the present work. When Fe^2+^ ions were introduced in the apatite structure, the partial charge distribution of the constituent atoms changed significantly (Table 3). Partial charges on Ca^2+^, H^+^ and P^5+^ ions generally increased with an increase in Fe^+2^ content, whereas the same on O(H) generally decreased (Table 3). This decline of the partial charges on O(H) atoms led to a reduced coulombic repulsion among OH^−^ groups arranged along *‘c’* axis, and eventually a contraction of the lattice parameter *‘c’* at higher Fe content (Table 1). This kind of fluctuations in the lattice parameters of doped HA have been observed in our previous experimental study^46^. On the other side, partial charges on O(P) atoms fluctuate as a function of dopant content (Table 3). The observed decrease in partial charges on O(H) resulted in the change in the attractive electrostatic interactions among Ca^2+^ and O(H) atoms, while the increased charges on Ca and H atoms enhanced the repulsive coulombic forces (Table 3). The effects of these changes got reflected in the decreasing nature of the tensile strength with dopant content (Table 2). It is also evident from Table 3 that the Fe^2+^ ions carry lesser partial charges than Ca^2+^ ions. Hence, the presence of Fe^2+^ ions gave rise to a weaker electrostatic attractive interaction among Fe^2+^ –PO_4_^3−^ or Fe^2+^ –OH^−^ ions compared to that among the Ca^2+^ –PO_4_^3−^ or Ca^2+^ –OH^−^ groups, present in pure HA. This also promotes the recorded decline in the tensile strength in doped HA.

As seen in Figs. 5, 6, and 7 and also in Table 2, the temperature also negatively impacts the tensile strength. With an increase in the temperature, the atomic/molecular vibration increases because of the enhanced thermal movement. This, in turn, affects the tensile strength in a negative manner. Other than the above-mentioned bond breakage at TS point, the deformations of several other bonds are also responsible for the linear response of HA under tensile loading (see Figs. 9, 10 and Fig. S1–S3 in Supporting information). The strength of these bonds also changed with doping content and temperature. The synchronized influence of these changes gave rise to the non-monotonous behaviour of the tensile strength.

**Figure 11:**
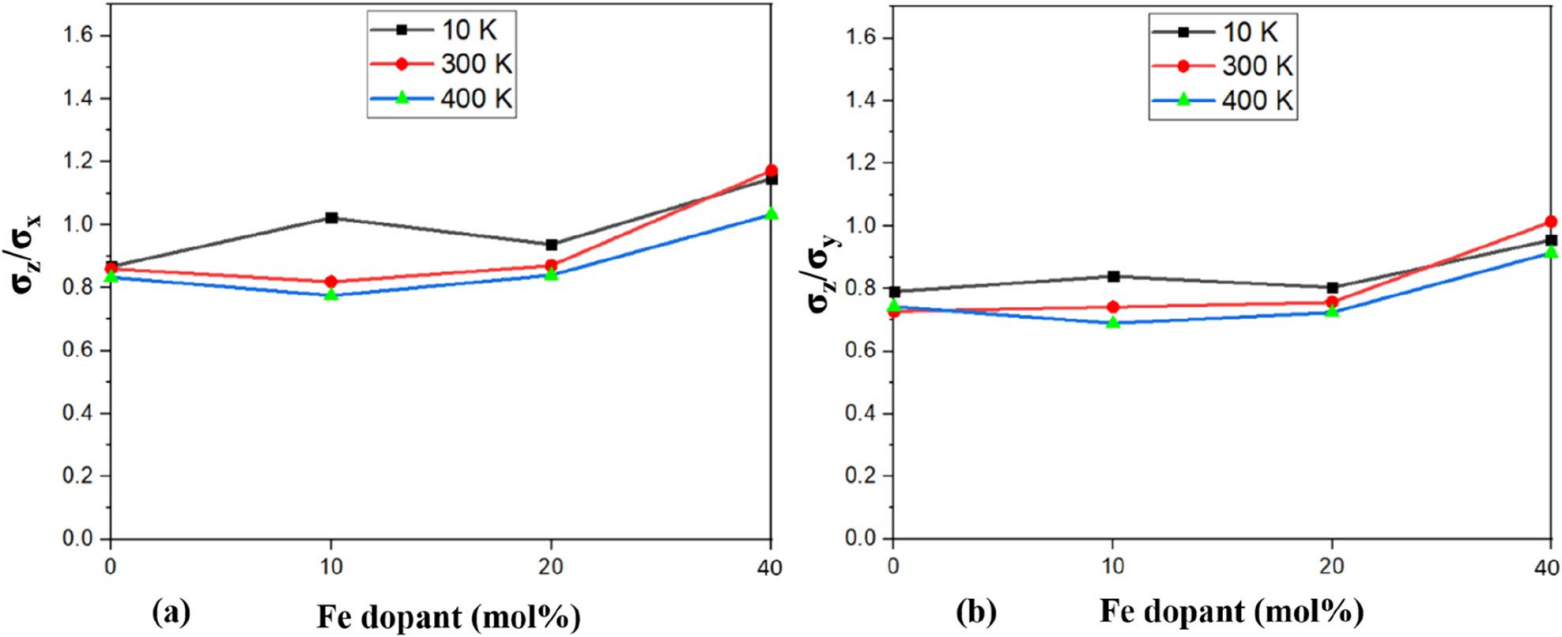
Tensile strength anisotropy (determined using MD simulation) at different temperatures and for doped HA single crystals.

In order to quantitatively analyze the differences in the mechanical response of doped HA in different loading directions, we have calculated the anisotropic variation in the tensile strength for undoped and Fe-doped HA, at different temperatures. In analyzing the anisotropic strength behaviour, Fig. 11 shows the ratios of tensile strength plotted as σ_z_/σ_x_ and σ_z_/σ_y_. A non-monotonous trend was observed for tensile strength (Fig. 11). A general observation is that the anisotropic nature of the tensile strength slightly decreased in the doped HA with an increase in temperature (Fig. 11). Like before, all these observations can be attributed to the altered partial charge distribution among the atoms in the presence of a foreign metal ion (Fe).

The anisotropic nature of the bone tissue is reported in the literature^50^. This arises from the preferential orientation of the collagen fibers and mineral crystals (calcium phosphate like HA) along the bone growth direction^51^. The anisotropic property of natural bone helps to maximize the stiffness and strength along the major load bearing axis, while keeping the bone mass minimum^52^. Hence, an ideal bone implant material should exhibit elastic anisotropy. HA is known to exhibit anisotropic mechanical response^50^, and our study establishes that the anisotropic nature of HA is retained after Fe^2+^ doping. Moreover, the elastic modulus of Fe^2+^-doped HA (data not shown) is close to that of cortical bone (7–30 GPa)^53^, which establishes its efficacy as bone implant material.

### Rearrangement of covalent bonds during tensile loading

To study the effects of the tensile force on the covalent bonds, bond strain and the change in vibrational bond energy have been calculated.

First, we analyze the changes in the characteristic properties of covalent bonds, (P–O) due to the application of the tensile force at both 10 K and 300 K. We have computed the bond strain and the effective change in its vibrational bond energy as a function of applied strain. These quantities are calculated using the following expressions,

3 $$\Delta {\text{r}} = {\text{r}} - {\text{r}}_{0}$$

4 $$\Delta {\text{E}} = 0.{5} \times {\text{k}}\left( {{\text{ r}}_{0}^{{2}} - {\text{ r}}^{{2}} } \right)$$

where k is the force constant and r_0_ is the equilibrium bond length.

**Figure 12:**
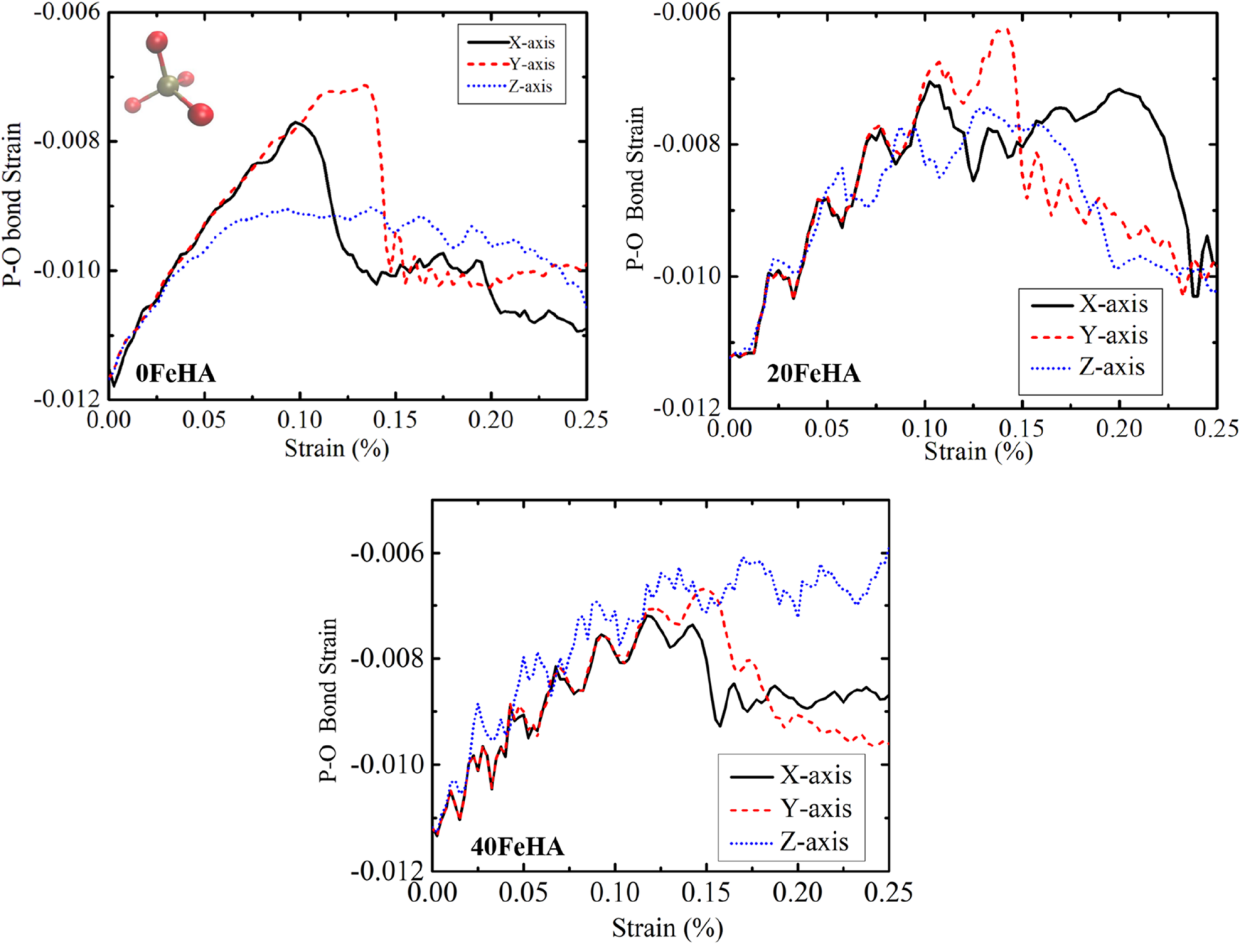
MD simulation analyzed dynamic changes in P–O bond strain with uniaxial tensile strain, for different doped HA at T = 10 K. Phosphate group is shown in the inset, with atomic colour code: O: red, P: golden-yellow. (Sample designation: xFeHA means x mol% Fe^2+^-doped HA).

**Figure 13:**
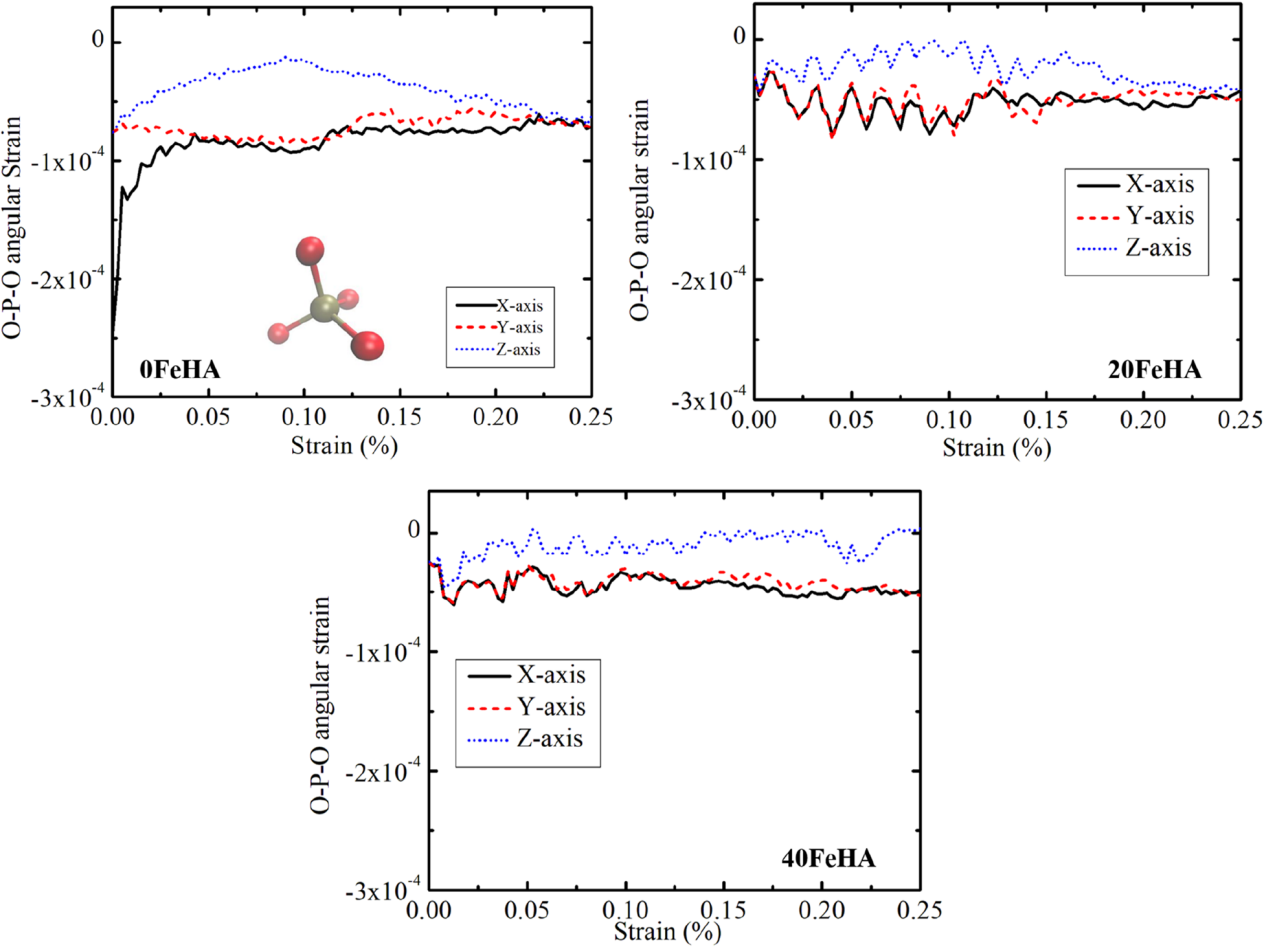
MD simulation analyzed variation in O–P–O angular strain during tensile loading for several Fe-doped HA at T = 10 K. Phosphate group is shown in the inset, with atomic colour code: O: red, P: golden-yellow. (Sample designation: xFeHA means x mol% Fe^2+^-doped HA).

The average bond strain of the P–O bonds, present in pure HA at 10 K has been presented in Fig. 12. The P–O bonds were found to be contracted at the beginning of the application of the tensile force and then stretched with the increment of the applied force (Fig. 12). After the TS point, the bonds again contracted and remained in that state for the rest of the simulation time (Fig. 12). In corroboration with the stress–strain curve (Figs. 5, 6, 7), the above-mentioned trend is most prominent when the force was applied along X and Y-axis. On the other hand, the O–P–O angle slightly decreased with the application of the tensile force, and then it maintained a steady state from 0.05% strain onward (Fig. 13).

**Figure 14:**
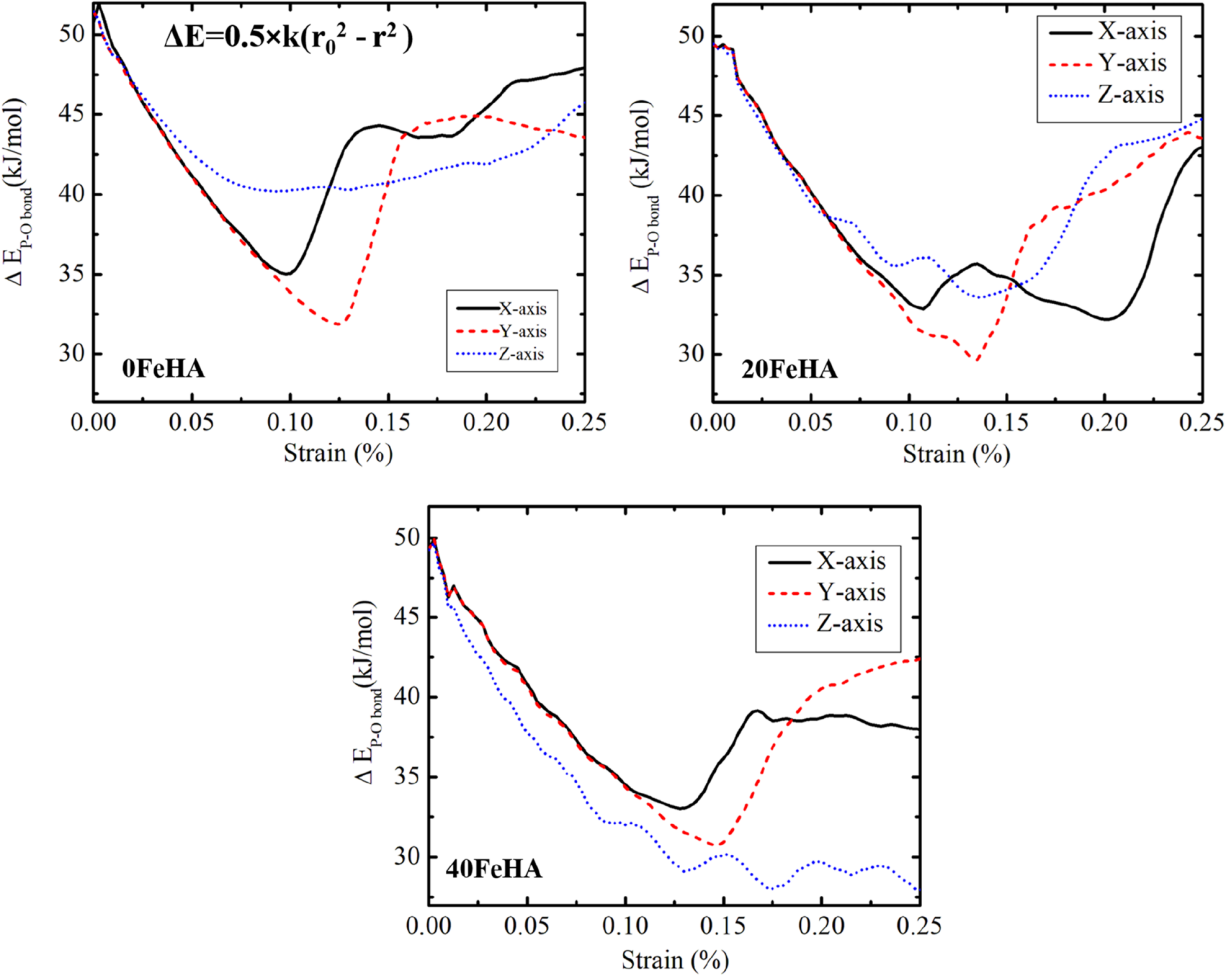
MD simulation analyzed change in vibrational energy of P–O bonds with uniaxial tensile strain, for various Fe-doped HA at T = 10 K. (Sample designation: xFeHA means x mol% Fe^2+^-doped HA).

The changes in the P–O vibrational bond energy at 10 K took place in complete harmony with the bond strain (Fig. 14). For X and Y-loading directions, the change was maximum initially and then reached minima near the TS point. Beyond that, the vibrational bond energy was again moved further away from its value at the equilibrium position, r_0_ (Fig. 14). The bond energy under stress was recorded to be closer to the equilibrium bond energy value near TS point, when the force was applied along Y-axis, compared to that for other loading directions (Fig. 14). In agreement with the stress–strain curve (Figs. 5, 6, 7), the observed pattern of the bond energy change under stress was significantly different for Z-axis (Fig. 14). The anisotropic nature of the HA crystal is the underlying reason behind the differences recorded in the change in bond energy under stress. Because of the different atomic arrangements along the various crystallographic directions, the P–O bonds were experiencing significantly divergent resultant forces, when exposed to the same external stress along the orthogonal loading direction. Such phenomenon results in distinct anisotropic behaviour, as presented in Fig. 14.

When the temperature was elevated to 300 K, the general trend of both the bond strain and change in bond vibrational energy for pure HA remains identical with additional oscillations (Fig. S6 in Supporting Information). This is attributed to the thermal motion at high temperature. At both 10 K and 300 K, the behaviour of P–O bond strain/bond energy of Fe-doped HA under external tensile stress remains mostly identical to that of pure HA at 10 K and 300 K, respectively (Fig. S7 in Supporting Information). The only significant change observed in P–O bond strain was for 40 FeHA at 10 K with loading direction along the Z axis (Fig. 12). Throughout the tensile loading, the P–O bonds were found to be expanded, probably because of the high Fe^2+^ content (Fig. S7 in Supporting Information). Similar behaviour of P–O bonds for 40 FeHA was absent at 300 K due to the thermal perturbation (Fig. S7 in Supporting Information).

The signature of the bond deformation with increased temperature can be found in the O–P–O angular strain in terms of oscillatory nature as well (Fig. S8 in Supporting Information). At 10 K, the angular strain became much smaller for Fe-doped HA, compared to pure HA (Fig. 13), whereas similar angular stain was recorded for each material at 300 K (Fig. S8 in Supporting Information).

**Figure 15:**
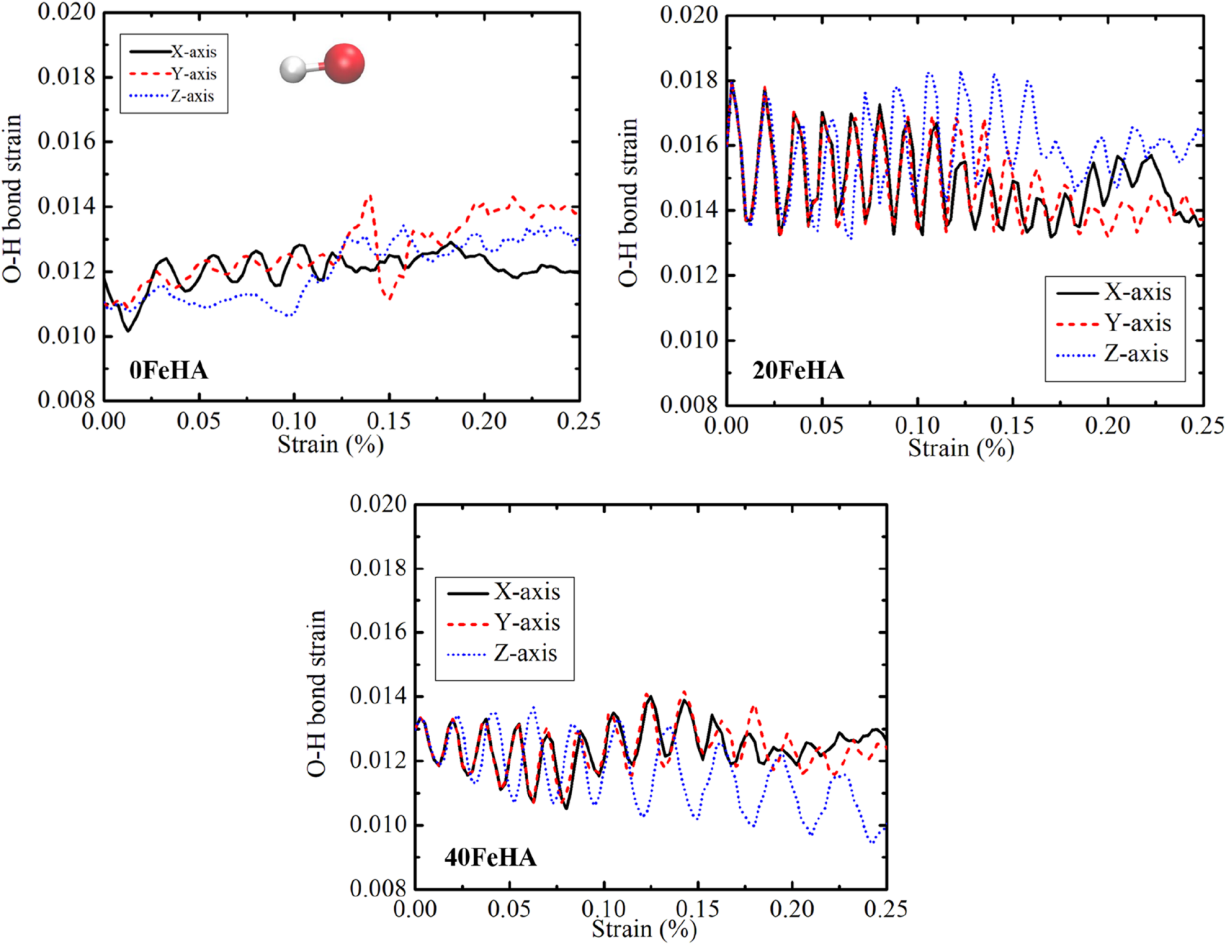
Oscillatory behaviour of O–H bond strain under tensile loading, as analyzed using MD simulation, for different Fe-doped HA at T = 10 K. OH group is shown in the inset, with atomic colour code: O: red, H: white. (Sample designation: xFeHA means  x mol% Fe^2+^-doped HA).

**Figure 16:**
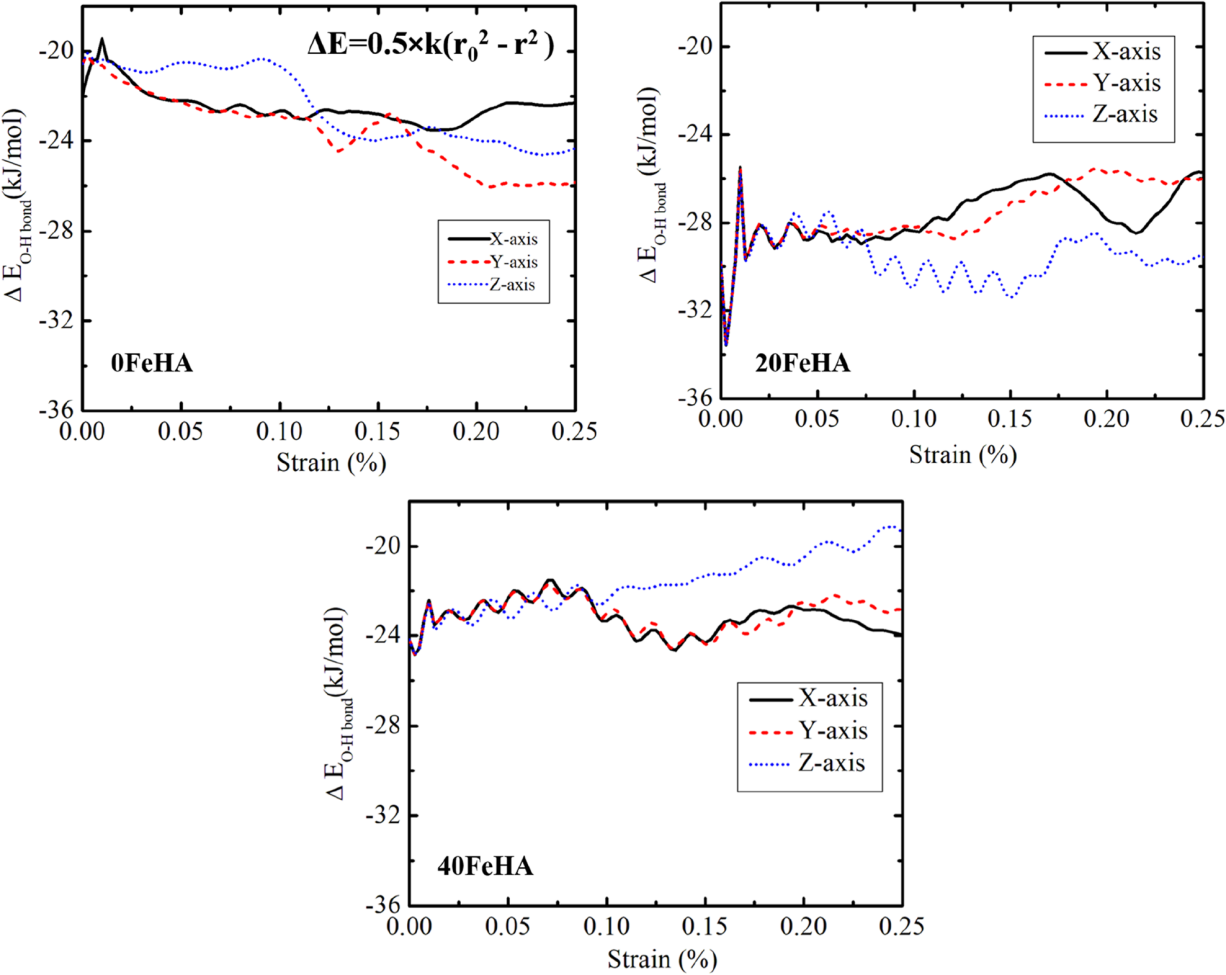
MD simulation analyzed changes in  vibrational energy of O–H bonds for Fe-doped HA at T = 10 K. (Sample designation: xFeHA means x mol% Fe^2+^-doped HA).

As far as the O–H bond strain of the pure HA is concerned, it exhibited oscillatory behaviour under the external stress at 10 K (Fig. 15), in corroboration with the change of O–H bond vibrational energy (Fig. 16). It is seen from the obtained atomistic trajectory during the tensile loading that the OH^−^ groups underwent orientational changes under the tensile stress. The oscillatory bond strain is a signature of the force experienced by the atoms of the hydroxyl groups during the orientational change. The frequency of oscillation increased in the presence of Fe^2+^ ions at 10 K, because of the altered interactions among the hydroxyl groups and metal ions (Fig. 15). The amplitude of oscillation was noticed to be higher in 20FeHA, compared to that in 40feHA. The probable reason behind such behaviour is the presence of unique Ca(1) column in 20FeHA, where Ca^2+^ and Fe^2+^ ions reside adjacent to each other along *‘c’* axis. Concomitantly, OH^−^ groups probably experienced more tensile forces, due to the presence of these heterogeneous atomic columns, which resulted in the more pronounced oscillatory character of O–H bond strain. For pure and Fe-doped HA, the amplitude of oscillation increased together with a decrease (reduction) in the oscillation frequency as well as with the increment of temperature, because of thermal motion (Fig. S9, see supporting information).

On the other hand, the oscillatory nature of the change in the O–H bond vibrational energy is more prominent at 300 K for 0FeHA and 20FeHA, because of the greater change in its bond length at higher temperatures (Fig. S9 and S10 in Supporting Information). For 40FeHA, the oscillatory nature is visible in both 10 K and 300 K, although the amplitude is larger at 300 K, due to greater (larger) thermal vibration (Fig. S10 in Supporting Information). Summarizing the results in this section, we can conclude that although the application of external tensile stress results in the change in both covalent bond length (Δr) and its corresponding vibrational energy (ΔE) in the apatite structure, such changes do not appear to result in the bond breakage. Moreover, the presence of dopant ions and the change in temperature also significantly influence the behaviour of the covalent bonds in apatite structure.

Most importantly, our study has provided an atomistic comprehension to the tensile response of single crystalline HA. On the experimental aspects, Gupta and co-workers performed a series of synchrotron-based studies to quantitatively analyze the deformation mechanism of natural bone, which is a composite of collagen and HA^54–56^. Since the elastic stiffness or strength properties largely depend on HA content in natural bone, we believe that the computational output from studies like the present study can be used further to validate the synchrotron-based experimental results. Also, synchrotron-based in situ study on single crystalline HA can be performed in future.

At the closure, our study shed light to the fracture mechanism of the single crystalline doped HA using a model system, which will be helpful to understand the planned experimental results. Similar in silico studies can be used to generate significant data for biomaterialomics approach^57^.

## Conclusions

In the present study, we have successfully implemented MD simulation as an effective computational platform to develop our foundational atomistic understanding of  the structural changes in Fe-doped HA and the dopant-dependent changes in ionic arrangement together with its impact on the mechanical response. Following key conclusions can be drawn from the present work.

- Between two crystallographically non-equivalent Ca sites, Ca(1) sites are slightly preferable over Ca(2) sites for Fe^2+^ substitution inside the apatite structure. The lattice parameters significantly change along the ‘*c’* axis after doping.
- The presence of Fe^2+^ ions inside the doped HA lattice restricts the chemical symmetry around the functional groups and this corroborates well with the changes in the number and intensity of the calculated IR bands.
- The tensile strength of HA decreases with Fe^2+^ doping because of the changes in the atomic charge distribution inside the crystal structure. The elastic modulus of Fe-doped HA is comparable with the natural cortical bone. An increase in temperature induces higher bond vibration and thermal movement, which significantly decreases the tensile strength of HA.
- The tensile deformation of Fe-doped HA at the atomistic level has been quantitatively analyzed using the dynamic changes in the major physical parameters of the covalent/ionic bond framework, including Ca–P distance, P–O bond strain, O–P–O angular strain, O–H bond distance with strain. Also, the temperature-dependent changes in tensile deformation have been analyzed by computing the dynamic changes in vibrational bond energy for O–H and P–O bonds.
- No significant breakage of the covalent bonds in the apatite structure is recorded during the tensile deformation. O–H bond strain exhibited oscillatory nature under tensile load, in a manner dependent on Fe^2+^ dopant content.

Taken together, the current study presents a molecular-level insight into the altered structure and mechanical behaviour and establishes the efficacy of MD simulation platform to gain deeper atomistic insights into the mechanical response of single crystalline doped HA.

## Supplementary Information

Supplementary Information.

## Data Availability

The data that support the findings of this study are submitted to the materials Cloud archive (https://archive.materialscloud.org/) with https://doi.org/10.24435/materialscloud:13-sa.
