# Supplementary material for: In silico study on probing atomistic insights into structural stability and tensile properties of Fe-doped hydroxyapatite single crystals
Source: Sci Rep. 2022 Nov 29;12:20576. doi: 10.1038/s41598-022-24904-0 (PMC9709045; doi:10.1038/s41598-022-24904-0)
Supplement: Supplementary file 1 — Supplementary Information. [file 41598_2022_24904_MOESM1_ESM.docx]

**Supporting information**

***In silico* study on probing atomistic insight into structural stability and tensile properties of Fe-doped hydroxyapatite single crystals**

**Subhadip Basu^1,⸸^, Shubhadeep Nag^2,⸸^, Nihal B Kottan^1,⸸^, Bikramjit Basu^1,3,*^**

^1^Materials Research Centre, Indian Institute of Science, Bangalore-560012, India

^2^ Solid State and Structural Chemistry Unit, , Indian Institute of Science, Bangalore-560012, India

^3^Center for Biosystems Science and Engineering, Indian Institute of Science, Bangalore-560012, India

**^⸸^Equal Contribution**

***Corresponding author;** e-mail: [bikram@iisc.ac.in (B.Basu)](mailto:bikram@iisc.ac.in%20(B.Basu))

**Table S1: Comparison between calculated and experimental IR spectra of undoped HA. Experimental data is taken from ref.**^1^

| **Peak Assignments** | **Computational (cm^-1^)** | **Experimental (cm^-1^)** |
| --- | --- | --- |
| **PO_4_^3-^ *v_4_* bands** | **532** | **567** |
| **PO_4_^3-^ *v_2_* bands** | **497** | **_** |
| **PO_4_^3-^ *v_3_* bands** | **1100-1041** | **1105-1100** |
| **PO_4_^3-^ *v_1_* bands** | **-** | **963** |
| **OH^-^ stretching bands** | **3601** | **3572** |


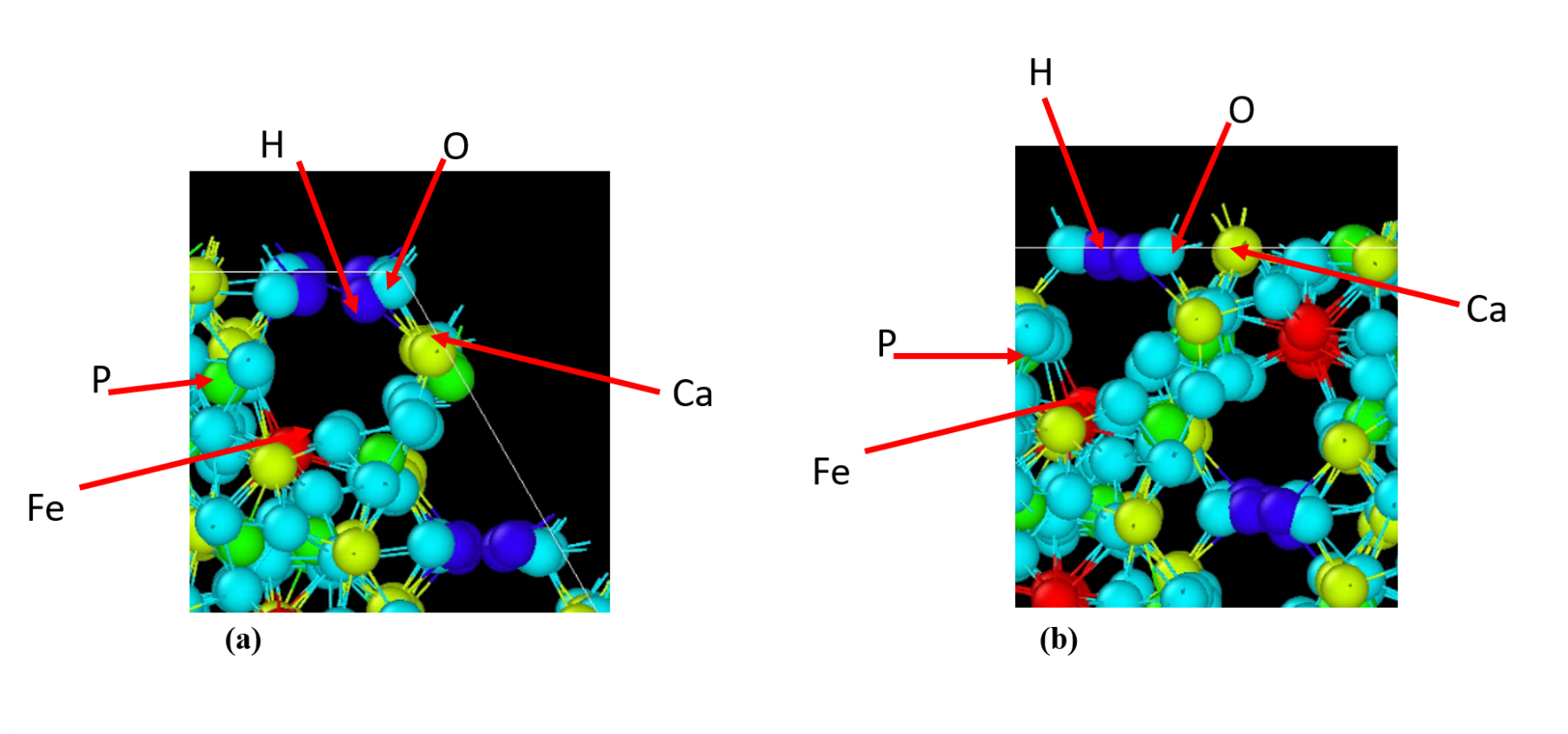


**Fig. S1:** Snapshot of 20FeHA at T = 300K at the UTS point on stress strain curve with loading direction along (a) X, and (b) Y axis. (Sample designation: xFeHA means x mol% Fe^2+^-doped HA).


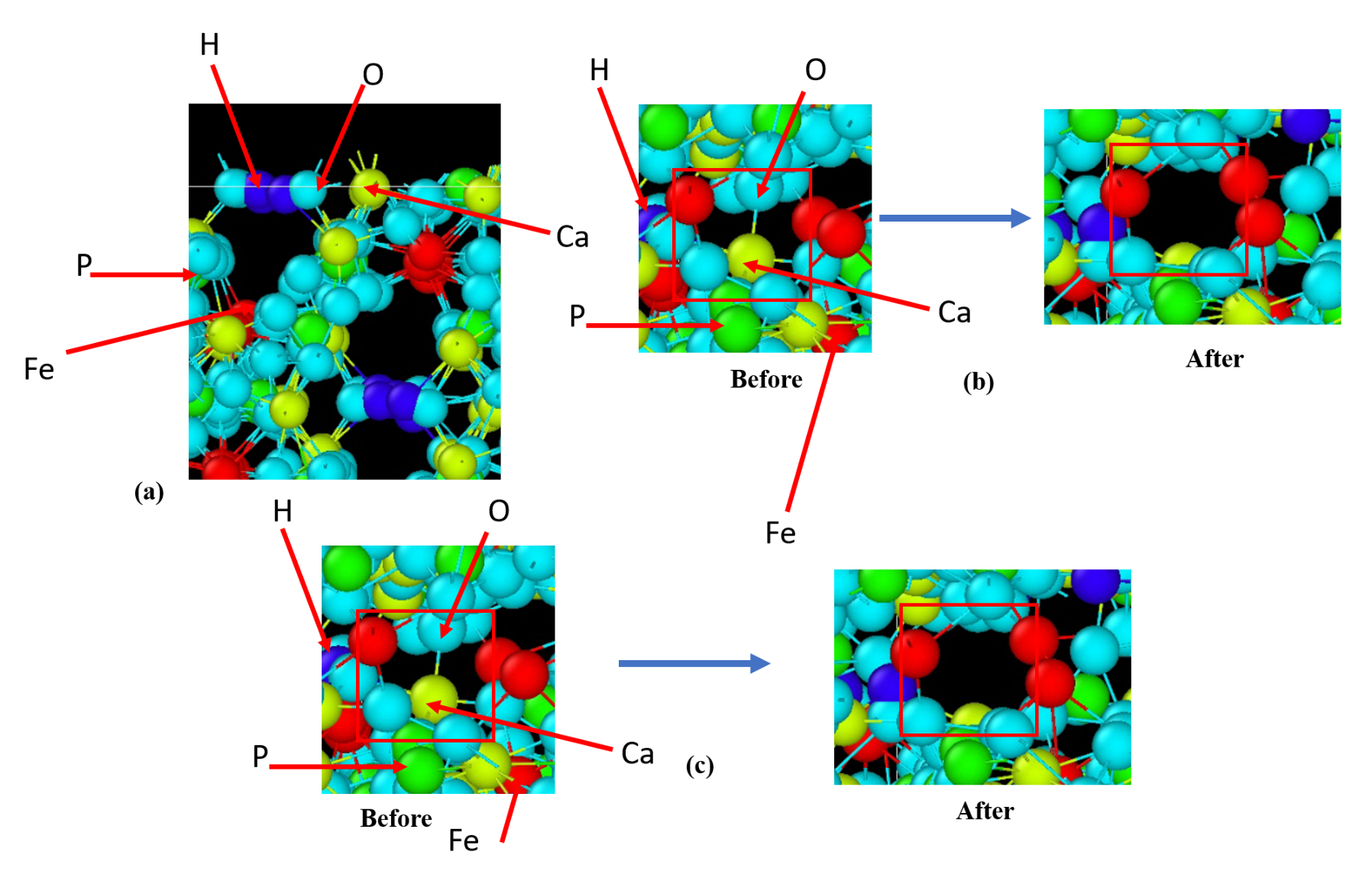


**Fig. S2:** Snapshot of 30FeHA at T = 300K at the vicinity of UTS point on stress strain curve with loading direction along (a) X, (b)Y, and (c) Z axis. “Before” and “After” indicates the state of the system prior and after bond breaking at UTS point. The region(s) marked by red square highlights broken bond(s). (Sample designation: xFeHA means x mol% Fe^2+^-doped HA).


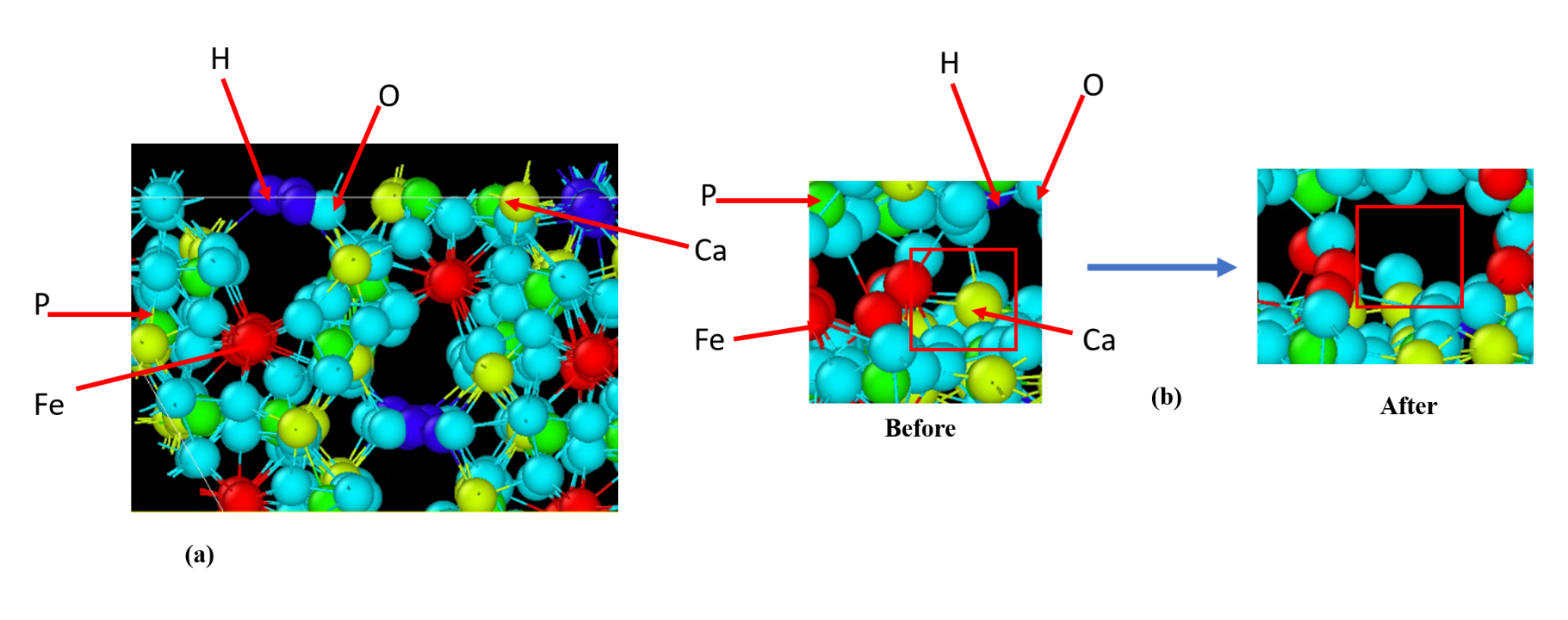


**Fig. S3:** Snapshot of 40FeHA at T = 300K at the vicinity of the UTS point on stress strain curve with loading direction along (a) X, and (b) Z axis. “Before” and “After” indicates the state of the system prior and after bond breaking at UTS point. The region(s) marked by red square highlights broken bond(s). (Sample designation: xFeHA means x mol% Fe^2+^-doped HA).


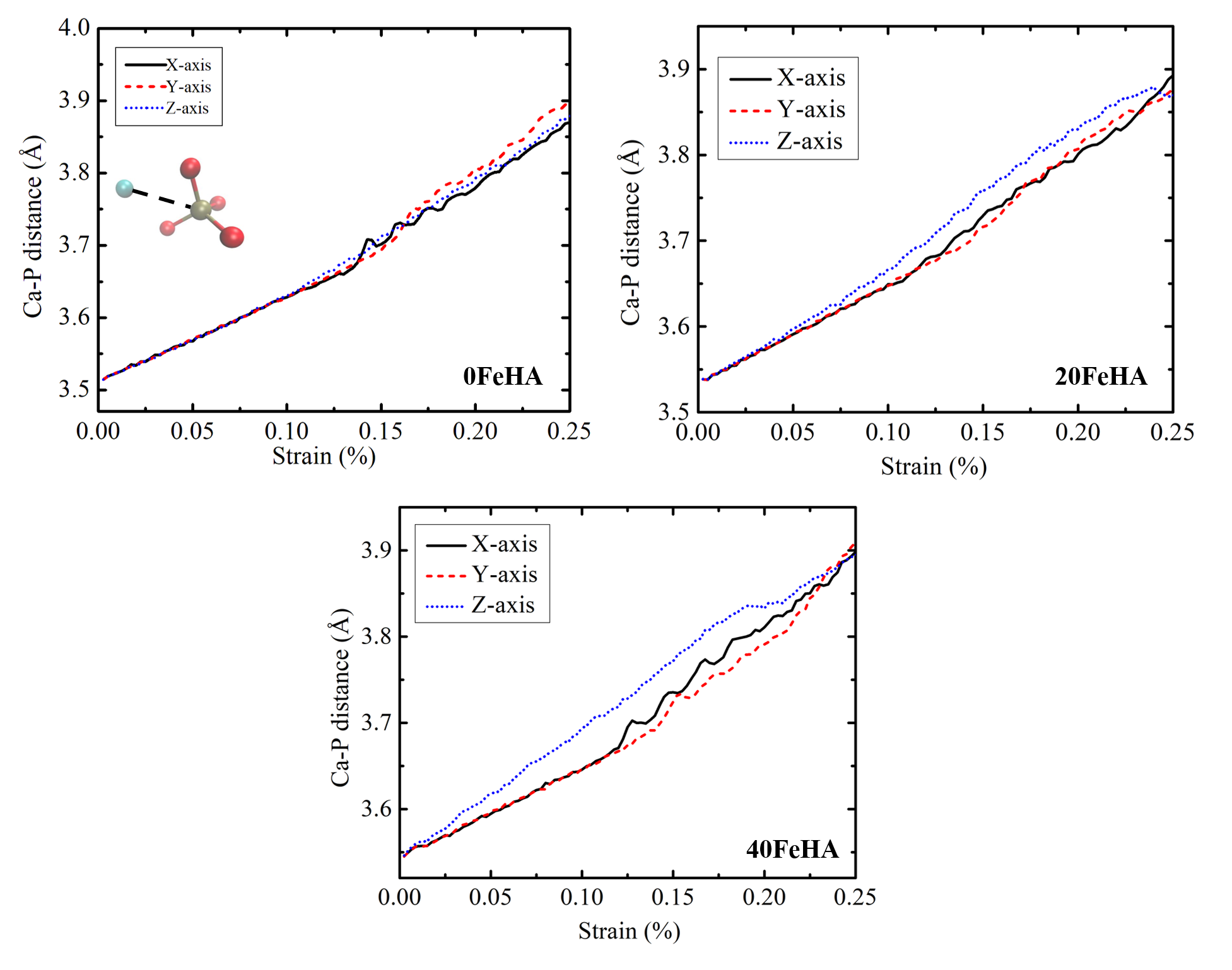
**Fig. S4:** Distance between Ca^2+^ and P^5+^ ions as function of uniaxial tensile strain, for different doped HA at T = 300 K. The distance has been defined schematically in the inset. Atomic colour code: Ca: pale blue, O: red, P: golden-yellow. (Sample designation: xFeHA means x mol% Fe^2+^-doped HA).


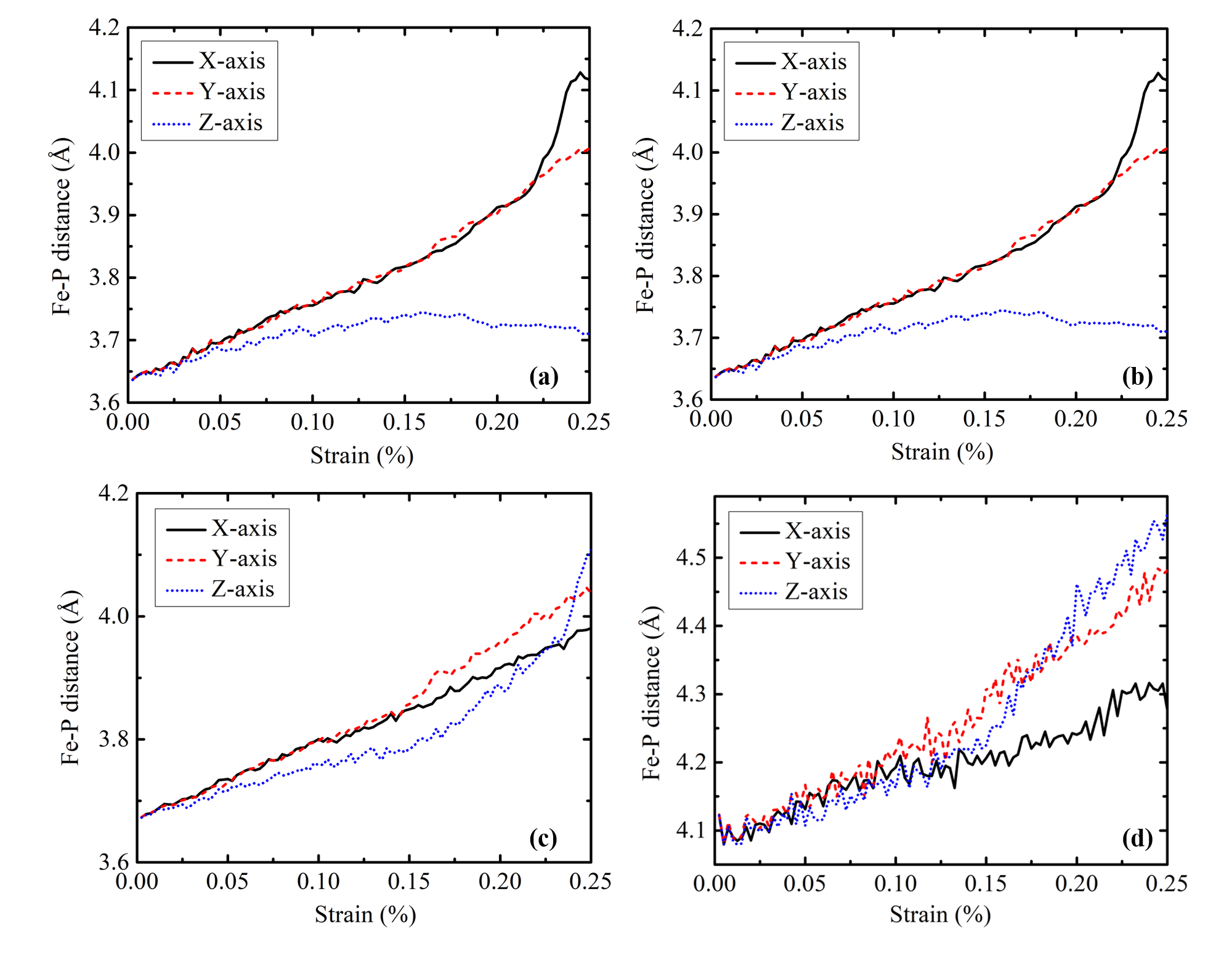


**Fig. S5:** Distance between Fe^2+^ and P^5+^ ions as function of strain, for (a) 20FeHA and (b) 40FeHA at T = 10 K. Same quantity has been shown for (c) 20FeHA and (d) 40FeHA at T= 300K. (Sample designation: xFeHA means x mol% Fe^2+^-doped HA).


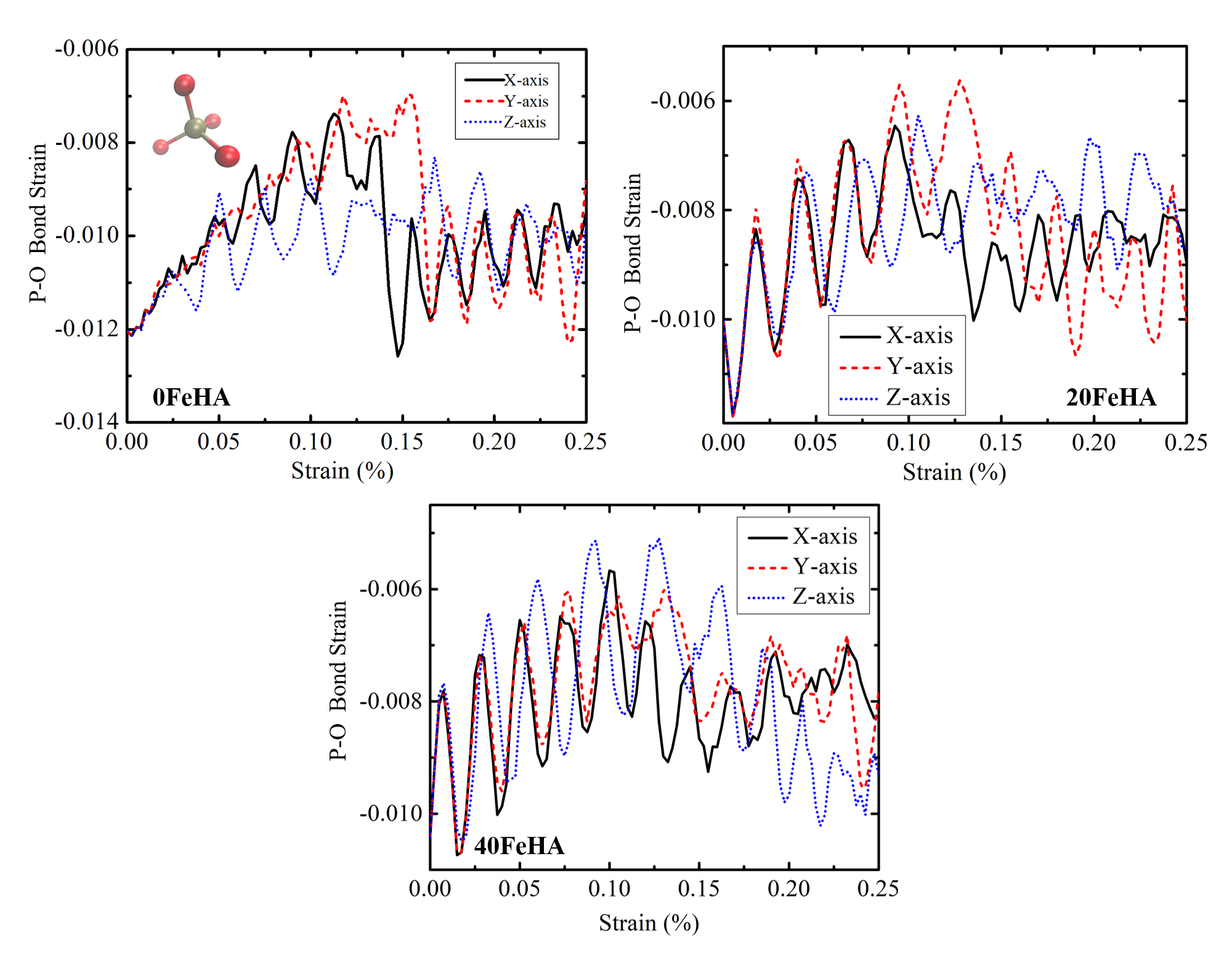
**Fig. S6:** Dependence of P-O bond strain on uniaxial tensile strain, for different doped HA at T = 300K. Phosphate group is shown in the inset, with atomic colour code: O: red, P: golden-yellow. (Sample designation: xFeHA means x mol% Fe^2+^-doped HA).


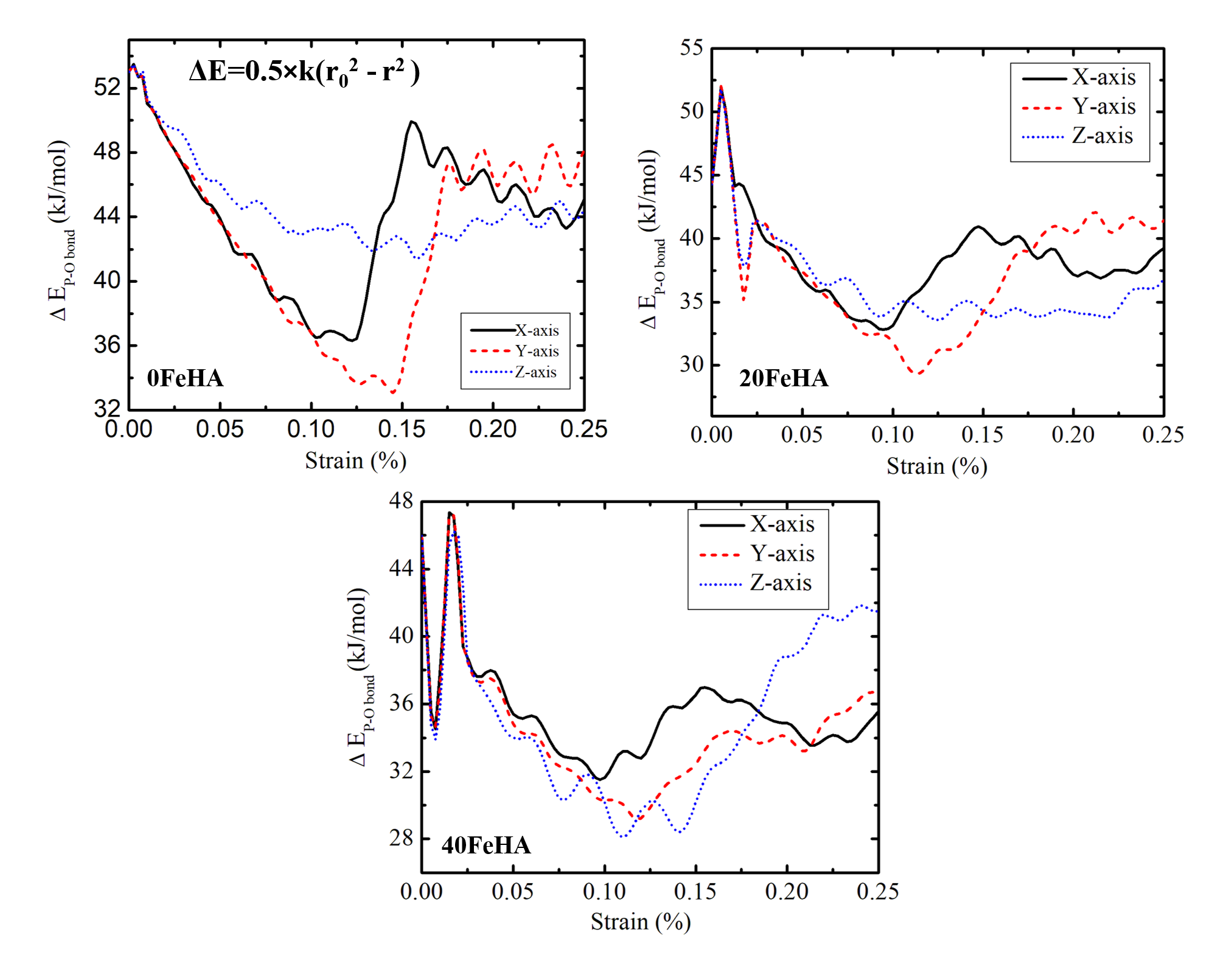


**Fig. S7:** Change in vibrational energy of P-O bonds with uniaxial tensile strain, for various Fe-doped HA at T = 300K. (Sample designation: xFeHA means x mol% Fe^2+^-doped HA).


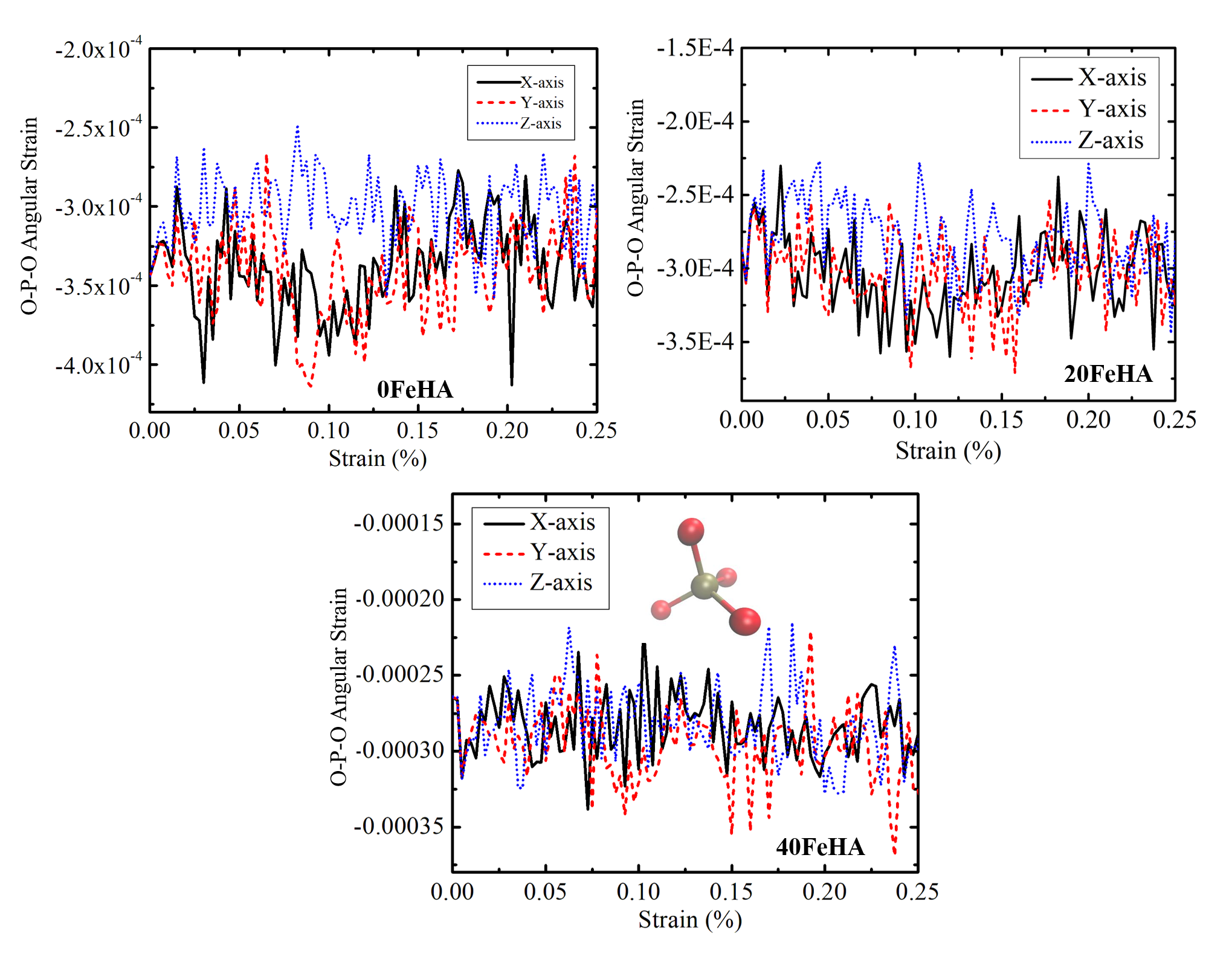


**Fig. S8:** Variation of O-P-O angular strain during tensile loading for several Fe-doped HA at T = 300K. Phosphate group is shown in the inset, with atomic colour code: O: red, P: golden-yellow. (Sample designation: xFeHA means x mol% Fe^2+^-doped HA).


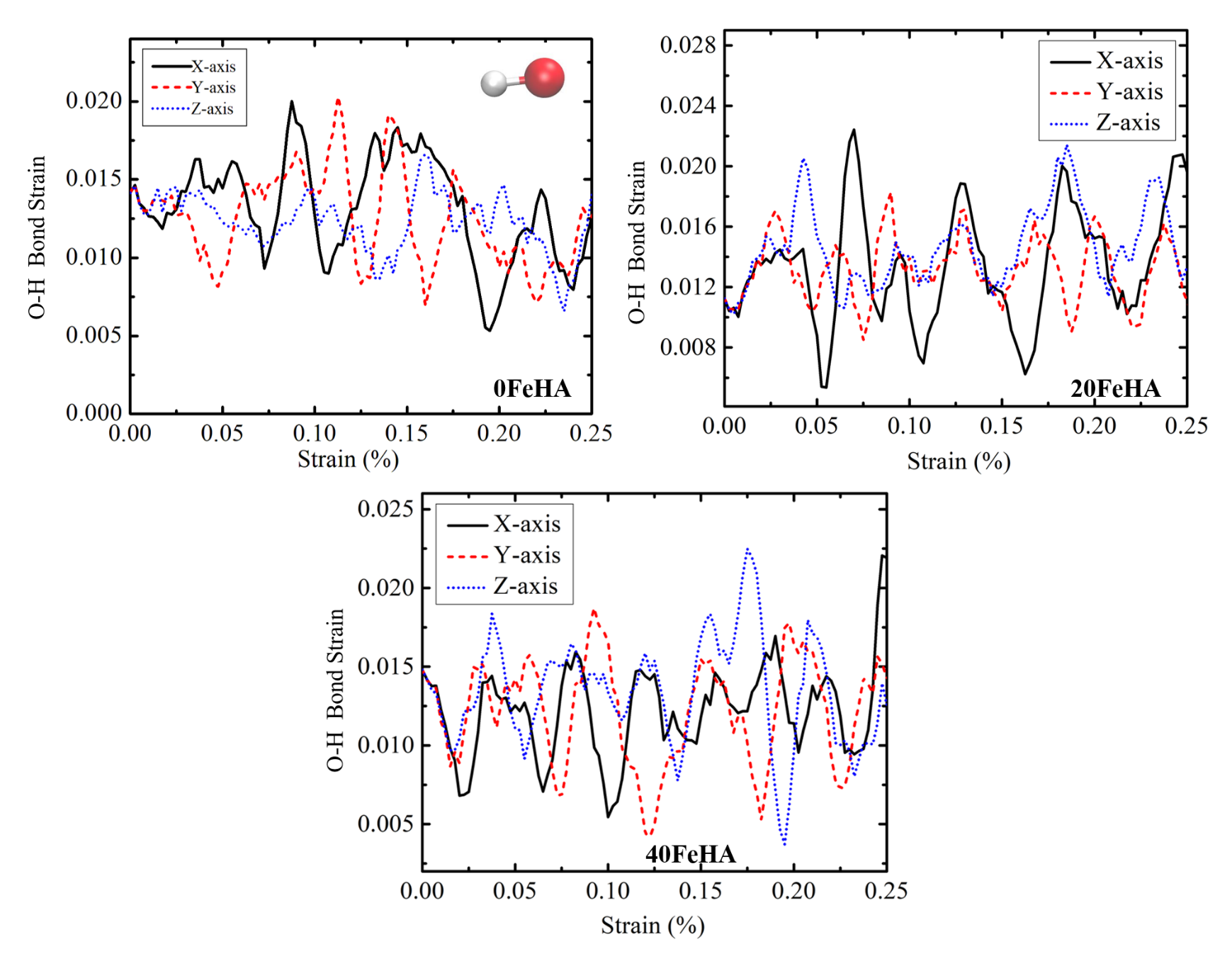


**Fig. S9:** Oscillatory behaviour of O-H bond strain under tensile force for different Fe-doped HA at T = 300K. OH group is shown in the inset, with atomic colour code: O: red, H: white. (Sample designation: xFeHA means x mol% Fe^2+^-doped HA).


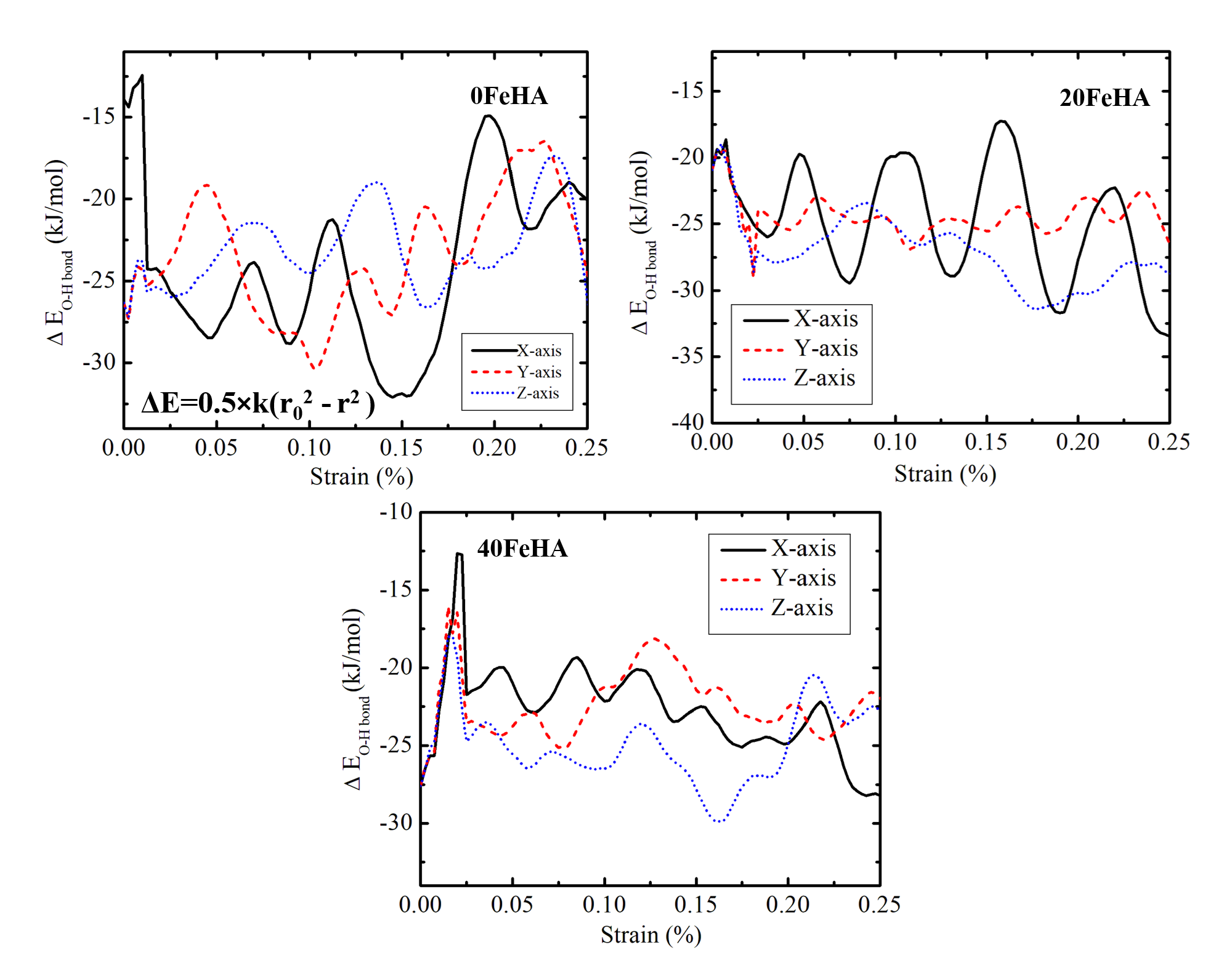


**Fig. S10:** Effects of tensile stress on vibrational energy of O-H bonds for Fe-doped HA at T = 300K. (Sample designation: xFeHA means x mol% Fe^2+^-doped HA).

**References**

1 S. Basu, A. Ghosh, A. Barui and B. Basu, (Fe/Sr) Codoped Biphasic Calcium Phosphate with Tailored Osteoblast Cell Functionality, *ACS Biomater. Sci. Eng.*, 2018, **4**, 857–871.
